# Multi‐Omics Signatures of Periodontitis and Periodontal Therapy on the Oral and Gut Microbiome

**DOI:** 10.1111/jre.70055

**Published:** 2025-11-27

**Authors:** Giacomo Baima, Shareef Dabdoub, Vivek Thumbigere‐Math, Davide Giuseppe Ribaldone, Gian Paolo Caviglia, Leonardo Tenori, Linda Fantato, Alessia Vignoli, Mario Romandini, Ilario Ferrocino, Mario Aimetti

**Affiliations:** ^1^ Department of Surgical Sciences University of Turin Turin Italy; ^2^ Division of Biostatistics and Computational Biology University of Iowa College of Dentistry Iowa City Iowa USA; ^3^ Department of Periodontics University of Iowa College of Dentistry Iowa City Iowa USA; ^4^ Division of Periodontology University of Maryland School of Dentistry Baltimore Maryland USA; ^5^ Department of Medical Sciences University of Turin Turin Italy; ^6^ Department of Chemistry “Ugo Schiff” University of Florence Florence Italy; ^7^ Ninth People’s Hospital Shanghai Jiao Tong University School of Medicine Shanghai China; ^8^ Department of Agriculture, Forest and Food Science University of Turin Turin Italy

**Keywords:** gut microbiome, metabolomics, metagenomics, multi‐omics, oral microbiota, oral–gut axis, periodontal diseases, periodontal treatment, periomedicine

## Abstract

**Aim:**

To characterize the impact of periodontitis and of Steps I–II of periodontal therapy on microbiome composition, function, and metabolic output across the oral and gut environments.

**Methods:**

A multi‐omics analysis was performed on saliva and stool samples collected from 50 systemically healthy individuals with and without Stage III–IV periodontitis. For participants with periodontitis, samples were analyzed both at baseline and 3 months after Steps I–II of periodontal therapy. High‐throughput whole metagenome sequencing was used to profile microbial taxa and functional genes, NMR‐based metabolomics profiled host–microbial metabolites. Single‐omic differential abundance analysis between healthy samples and periodontitis samples was performed with MaAsLin2, while analysis between pre‐ and post‐treatment was conducted with timeOmics. Variable selection and subsequent supervised multivariate analysis to determine group‐separating markers utilized multi‐level sparse Partial Least Squares Discriminant Analysis (sPLS‐DA) through mixOmics. KEGG pathway enrichment was analyzed using clusterProfiler, whereas multi‐omic data integration was performed with multi‐block Partial Least Squares regression analysis.

**Results:**

Periodontitis was associated with significant compositional and functional changes in both saliva and stool, with increased abundance of pathobionts and loss of health‐associated taxa in both niches. A subset of species was shared across oral and gut habitats, with detectable differences across clinical groups. As functional potential, periodontitis enriched microbial pro‐inflammatory pathways (lipopolysaccharide biosynthesis, bacterial motility) and depleted beneficial short‐chain fatty acid (SCFA)‐ and vitamin‐producing functions. Metabolomic profiles revealed reduced SCFAs and amino acids in periodontitis, with elevated pro‐inflammatory metabolites (succinate, trimethylamine) in both saliva and stool. Following therapy, microbial communities and their metabolic output partially reverted toward health‐associated profiles, particularly in saliva. Stool samples showed subtler but consistent shifts, including a decrease in some typically oral species and decreased succinate and methylamine and restoration of amino acid and SCFA‐related metabolites.

**Conclusions:**

Periodontitis is associated with coordinated microbial and metabolic signatures across the oral and gut environments. Non‐surgical periodontal therapy promotes partial ecological restoration in both niches, supporting the view of oral health as a modifiable target for influencing systemic microbial homeostasis.

**Trial Registration:**

ClinicalTrials.gov identification number: NCT04826926

## Graphical abstract

This multi‐omics study integrates shotgun metagenomics and NMR metabolomics to reveal coordinated microbial and metabolic signatures of periodontitis across the oral–gut axis. Non‐surgical periodontal therapy partially restored ecological and functional balance, supporting oral health as a modifiable driver of systemic microbial homeostasis.

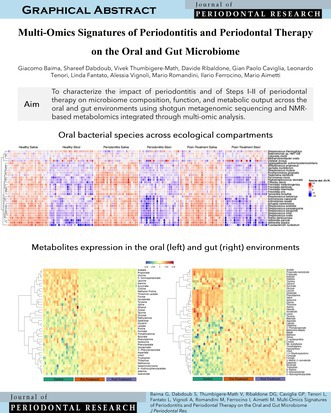

**Summary**

- Scientific background
  ○ Oral bacteria and periodontitis have been increasingly implicated in gut microbiota alterations, particularly in preclinical models. However, the extent to which periodontitis disrupts the oral–gut microbial and metabolic axis, and whether these disturbances can be reversed by periodontal therapy, remain largely unknown in humans.
- Added value of this study
  ○ This study presents the first high‐resolution, longitudinal multi‐omics characterization of oral and gut microbiomes and metabolomes in systemically healthy individuals with and without periodontitis. It reveals distinctive taxonomic, functional, and metabolic signatures associated with disease and demonstrates that Steps I and II of periodontal therapy can positively influence these alterations.
- Clinical implications
  ○ These findings highlight the potential utility of salivary and fecal biomarkers in distinguishing periodontal health from disease and in monitoring responses to periodontal treatment. More broadly, the results support the role of periodontal care as a means to modulate systemic microbial homeostasis—suggesting that treating periodontitis may influence systemic health not only by controlling local inflammation, but also by reshaping microbial communities along the oral–gut axis.

## 1 Introduction

The human microbiome is a complex and dynamic ecosystem composed of bacteria, viruses, archaea, and eukaryotic microorganisms that co‐exist with the host in a symbiotic relationship, forming what is often referred to as a “superorganism” [1]. The metabolic outputs of this microbial community—including proteins and small molecules—play an essential role in regulating both local and systemic physiological functions [2]. Among the most influential microbial habitats are the oral and gut microbiomes, each of which contributes to local and systemic health maintenance, but also plays a pivotal role in disease pathogenesis when dysbiosis occurs [3, 4].

Periodontitis is a highly prevalent chronic inflammatory disease characterized by a dysbiotic subgingival biofilm and a dysregulated host immune‐inflammatory response [5, 6, 7, 8], in systemically and/or environmentally susceptible individuals [9, 10, 11]. While traditionally considered a localized oral disease, periodontitis has been increasingly linked to systemic conditions through several mechanisms, also involving microbial translocation [12, 13, 14, 15, 16, 17, 18, 19, 20]. Emerging evidence also suggests that oral dysbiosis may influence gut microbial composition, supporting the existence of a microbial axis between the mouth and the gut [21, 22, 23, 24, 25, 26, 27]. One plausible mechanism underlying this axis is the enteral translocation of oral pathobionts through the continuous swallowing of saliva, which may lead to the establishment of oral‐derived pathobionts in the gut, although the hematogenous route can also be involved [28, 29].

However, whether a microbial or metabolic signature of periodontitis can be detected in the gut, and whether such signals are modifiable through periodontal treatment, remains largely unexplored. To date, most studies examining the oral–gut microbial axis in periodontitis have indeed employed 16S rRNA gene sequencing, limiting taxonomic resolution and failing to capture functional potential [30, 31, 32, 33, 34]. In contrast, shotgun metagenomics enables species‐ and strain‐level identification and allows for in‐depth exploration of microbial gene content and function [35, 36, 37, 38]. When paired with metabolomics, multi‐omics approaches can provide a comprehensive picture of both microbial ecology and its biochemical impact on the host [39, 40].

Despite these technological advancements, no human studies have applied multi‐omics strategies to investigate functional changes in both the oral and gut microbiota associated with periodontitis. Moreover, the role of salivary and fecal metabolomes—encompassing microbial metabolites, dietary derivatives, and host‐modified compounds—remains poorly defined [41, 42]. This study addresses these knowledge gaps by applying shotgun metagenomics and NMR metabolomics to examine the impact of periodontitis and the Steps I–II of periodontal therapy on microbiome composition, function, and metabolic output across the salivary and fecal environments.

## 2 Methods

The study protocol was approved by the Institutional Ethical Review Board at the University of Turin (protocol number 00066/2021), and written informed consent was obtained from all participants. This manuscript is reported in accordance with STROBE guidelines.

### 2.1 Study Population

Patients with generalized Stage III or Stage IV periodontitis were consecutively recruited from individuals seeking oral health consultation at the C.I.R. Dental School, University of Turin (Italy), between March 2021 and September 2022. Age‐ and gender‐matched, periodontally healthy participants were recruited as controls. Details for subjects' recruitment, sample collection and periodontal therapy have been reported in a previous publication reporting on 16S rRNA results [32].

Exclusion criteria, both in cases and controls, included: having < 20 teeth; diagnosis of systemic diseases, including diabetes mellitus and gastrointestinal disorders; a BMI of < 18.5 or ≥ 30 kg/m^2^; food allergies; current smokers; received periodontal treatment within the previous 12 months; consumption of antibiotics, probiotics or proton pump inhibitors (PPIs) within the previous 3 months; pregnancy or lactation; ongoing medications; or systemic antimicrobial use between baseline and 3‐month follow‐up. Inclusion criteria for the control group included: a full‐mouth bleeding score (FMBS) < 30%, no sites with interdental clinical attachment loss (CAL) > 1 mm at two non‐adjacent teeth, and no probing pocket depth (PPD) ≥ 6 mm (PPD = 4–5 mm due to pseudopockets were not exclusionary).

### 2.2 Clinical Procedures

All participants underwent a comprehensive periodontal examination by one calibrated examiner (G.B.). The presence of plaque (PI), bleeding on probing (BoP), PPD, and CAL was recorded at six sites per tooth using a manual probe (PCP UNC 15, Hu‐Friedy, Chicago, IL). Intra‐examiner reproducibility was assessed by taking replicate measurements on the same 10 participants with a 24‐h interval between the first and second recordings. The percentage of agreement within 1 mm of PPD and CAL ranged between 94% and 97%. During the first visit, all participants completed a dietary questionnaire (Italian EPIC Food Frequency Questionnaire) [43].

Periodontitis patients subsequently received Steps I–II of periodontal therapy according to EFP S3‐level clinical practice guideline [44]. Briefly, after a behavioral phase focused on oral hygiene instruction and the management of systemic risk factors, all patients underwent quadrant‐wise subgingival instrumentation (one session per week). Necessary treatments for caries and endodontic lesions were also completed prior to periodontal therapy [45, 46]. Follow‐up clinical evaluation, questionnaire administration, and sample collection occurred 3 months post‐therapy.

### 2.3 Saliva and Fecal Samples Collection

Within 3 days after their dental examinations, patients returned to provide saliva and fecal samples. Patients were instructed to avoid food, sugary drinks, caffeine, toothpaste, and mouthwashes the morning of collection [47]. Unstimulated saliva (~4 mL) was collected chair‐side between 8:00 and 10:00 AM. On the same appointment, patients delivered fecal samples that were self‐collected on the same morning using a polypropylene spoon into sterile fecal collection tubes (3 tablespoons of ~10 g). Saliva and fecal samples were then stored at −80°C until analyses.

### 2.4 Metagenomics and Bioinformatics Analysis

DNA was extracted from saliva and fecal samples using the QIAamp Power Fecal Pro DNA Kit (Qiagen, Milan, Italy), in accordance with SOP07 guidelines developed by the International Human Microbiome Standard Consortium (www.microbiome‐standards.org). The DNA concentration was quantified using the QUBIT dsHS kit and standardized to 5 ng/μL. Shotgun metagenomic sequencing was performed on an Illumina platform, generating paired‐end 150‐bp reads.

Raw sequences were first mapped against the draft genome of *H. sapiens* GRCh38, to remove human reads, using Bowtie2 (v.2.4.4) [48] in end‐to‐end sensitive mode. Non‐host sequences were quality filtered with AdapterRemoval (adapter, *Q* < 20, reads < 50 bp) [49]. Taxonomic assignment was performed using Woltka [50] v0.1.7 with Bowtie2 v2.4.4 to map human‐screened reads against the Human Oral Microbiome Database (HOMD) v9.15 for saliva samples and the Unified Human Gastrointestinal Genome (UHGG) database v2.0.2 combined with HOMD for stool samples. Gene annotation against the KEGG database was performed by HUMAnN3 with default parameters [51].

### 2.5 Metabolomics Analysis

Frozen saliva samples were prepared as previously described [52, 53]. Because fecal matter is highly heterogeneous and rich in macromolecules that compromise spectral resolution and reproducibility, from each stool sample fecal water was extracted at a ratio of 1:2.5 (g/mL, weight of feces to buffer volume) in potassium phosphate buffer. Frozen fecal water samples were thawed at room temperature and centrifuged. Subsequently, 300 μL of the supernatant was diluted with 600 μL of D_2_O, and centrifuged. 600 μL of the supernatant was transferred into a 5 mm NMR tube for analysis.

NMR spectra for both salivary and fecal water samples were acquired using a Bruker 600 MHz spectrometer (Bruker BioSpin) operating at 600.13 MHz proton Larmor frequency. The Carr–Purcell–Meiboom–Gill (CPMG) one‐dimensional spin–echo sequence (Bruker sequence cpmgpr1d) [52] was applied to detect metabolite signals using 64 scans for saliva samples and 128 scans for fecal water samples. Metabolite signals were manually assigned in processed spectra by using Chenomx NMR suite 12.0, freely available databases, and published literature (when available). Quantification (in arbitrary units) was performed by integration using an R script in‐house developed. To account for variations in water content, total spectral area normalization was applied to metabolite concentrations. A detailed description of sample preparation procedures and NMR methods is provided in the Appendix S1.

### 2.6 Data and Statistical Analysis

#### 2.6.1 Overview

We analyzed microbial community changes between pre‐treatment (T0) and post‐treatment (T1) timepoints using a multi‐omics approach that integrated taxonomic profiles, metagenomic functions, and metabolomic data. Control samples were collected at both timepoints for comparison. All computational analyses were performed using R version 4.4.3.

#### 2.6.2 Microbial Diversity Analysis

Alpha diversity, which measures diversity within individual samples, was assessed using two complementary metrics. The Shannon‐Wiener index captures both the number of different microbes present and how evenly distributed they are, while Observed Species provides a simple count of unique microbial taxa detected. These metrics were calculated using *phyloseq* (v1.50.0) and *vegan* (v2.7–1). To account for repeated sampling from the same individuals, we compared pre‐ and post‐treatment alpha diversity using generalized linear mixed models (GLMM) implemented in *lme4* (v1.1–37). This approach treats each individual as a random effect, controlling for person‐to‐person variation. Standard linear models were used for all other comparisons. For Observed Species counts, we used negative binomial models to handle the count‐based nature of this data. Specifically, we employed negative binomial GLMM through *glmmTMB* (v1.1.11) for paired pre/post comparisons and negative binomial generalized linear models via *MASS* (v7.3–65) for other comparisons. Model adequacy was verified using the check_overdispersion function from the performance package (v0.15.0) to ensure our count models appropriately handled data variability.

Beta diversity, which quantifies pairwise differences in microbial composition between samples, was calculated using Bray‐Curtis distance on Cumulative Sum Scaled (CSS) count data to measure community dissimilarity based on abundance. Statistical differences between groups were tested using PERMANOVA (Permutational Multivariate Analysis of Variance) via the adonis2 function in *vegan*, which determines whether group‐wise clustering differs significantly between groups by comparing group centroids. Testing for longitudinal beta diversity differences was determined using linear mixed effects models via the QIIME2 v2025.7 longitudinal linear‐mixed‐effects module.

Core taxa (those present in at least 80% of samples) were calculated using the *compute_core_microbiome* command in QIIME (v1.9) [54] and rendered to a table using the *PhyloToAST* (v1.4) [55] *core_overlap_plot* command. A subset of oral commensals and pathogens from the core species was selected to compare abundance across the oral and gut niches and visualized using CLR‐transformed counts with the *ComplexHeatmap* (v2.24.1) Bioconductor package for R.

#### 2.6.3 Differential Abundance Testing

We identified microbes, genes, and metabolites that changed significantly between conditions using complementary approaches. For between‐group comparisons of healthy subjects and periodontitis patients (pre‐ and post‐treatment separately) we used MaAsLin2 [56] (v1.22.0) to apply generalized linear models to centered log‐ratio (CLR) transformed (to account for compositionality) abundance data. For longitudinal analysis of T0 to T1 changes, we employed timeOmics (v1.20.0) [57], which is specifically designed for time‐series data using mixed models via the Linear Mixed Model Splines framework (lmms package v1.3.3). Briefly, a mixed model is fit to each observation with a *p*‐value cutoff of 0.05 and an overall FDR correction of *q* < 0.1. The CLR transformation addresses the compositional nature of microbiome data, where increases in some features necessarily decrease the relative abundance of others.

#### 2.6.4 Supervised Single‐Omic Analysis

We used the mixOmics [58] R package (v6.30.0) to identify microbial features and metabolites that best distinguish between treatment states. The analysis employed sparse Partial Least Squares Discriminant Analysis (sPLS‐DA), a method that selects the most informative features separating groups in high‐dimensional data with more predictors than samples. The best set of features was selected via 10‐fold cross validation (50 repeats) and the *max.dist* metric for estimating classification error rate and a significance threshold of *p* < 0.01 required to determine error rate improvement over a grid from 1 to 50 features. To properly analyze our repeated‐measures design where the same individuals were sampled at T0 and T1, we first extracted within‐individual variation using the *withinVariation* function [59]. This preprocessing step removes individual‐specific baseline differences, allowing sPLS‐DA to focus on ‐omic features most responsible for differences across all three groups (T0, T1, Control) simultaneously.

#### 2.6.5 Functional Analysis

Using the genes identified as significantly differentially abundant following treatment, we split them into those with decreased abundance and increased abundance. The magnitude of change for each gene was used as input to iPath 3 [60] to create KEGG pathway visualizations. To provide background reference, iPath 3 was also provided with the core genes (present in at least 60% of samples, calculated as above with taxa) from healthy subjects. Pathway enrichment analysis was performed using clusterProfiler (v4.14.6) and enrichplot (v1.26.6) to identify biological pathways significantly altered by treatment.

#### 2.6.6 Multi‐Omic Data Integration

To understand relationships between changes in microbes, genes, and metabolites, we performed multi‐block Partial Least Squares regression in canonical mode using *mixOmics* [61]. Results were visualized using correlation circle plots generated by the *plotVar* function, which display how strongly different features correlate with treatment response. Features with a correlation of rho < 0.6 were filtered out. This integrated analysis reveals coordinated changes across biological layers, providing insight into the mechanisms underlying treatment effects.

### 2.7 Sample‐Size Justification

Power simulations for PERMANOVA on Bray–Curtis/UniFrac distance matrices indicated that ≥ 20 subjects per group would have provided > 90% power (*α* = 0.05) to detect small between‐group effects (*ω* ^2^ ≈ 0.008), while 10 subjects would have sufficed for moderate effects (*ω* ^2^ ≈ 0.02) [62]. In addition, recent guidelines for untargeted ^1^H‐NMR metabolomics recommend > 20 biological replicates per arm to ensure model stability [63]. To exceed both thresholds and to allow for possible attrition at follow‐up, we enrolled 25 participants in each study group.

## 3 Results

A total of 50 individuals (25 with Stage III–IV periodontitis and 25 controls without periodontitis) were included. For metagenomic analyses, the whole set of 75 saliva and 75 fecal samples was selected: 25 samples of each type from each group (controls, periodontitis pre‐treatment, and periodontitis post‐treatment). Metabolomic analyses were performed on the same specimens used for metagenomics; however, in a subset of cases, the available sample volume was insufficient for NMR profiling. As a result, 66 saliva samples were analyzed (23 from controls, 23 from subjects with periodontitis pre‐treatment, and 20 post‐treatment samples), along with 61 stool samples (19 from controls, 22 pre‐treatment, and 20 post‐treatment).

### 3.1 Population Characteristics and Response to Periodontal Therapy

The study groups were well balanced in terms of age, sex, BMI, diet, and smoking status (Table 1 and Table S1). As expected, the periodontitis group displayed significantly higher levels of FMBS, full‐mouth plaque score (FMPS), and mean number of PPD. In this group, Steps I–II of periodontal therapy were effective in improving all the considered clinical parameters at 3 months (*p* < 0.001).

**TABLE 1:** Characteristics of the study population.

Variable	Periodontal health (*n* = 25)	Periodontitis pre‐therapy (*n* = 25)	*p*	Periodontitis post‐therapy (*n* = 25)	*p*
Age (years), mean (SD)	53.7 (9.9)	54.1 (10.4)	> 0.05	/	/
Gender, *n* (%)					
Females	13 (52.0)	14 (56.0)	> 0.05	/	/
Males	12 (48.0)	11 (44.0)			
BMI (kg/m^2^), mean (SD)	23.8 (2.8)	26.5 (3.6)	> 0.05	/	/
FMPS	24.9 (18.3)	73.1 (20.1)	< 0.001*	26.6 (12.5)	< 0.001**
FMBS	17.1 (15.5)	72.1 (19.5)	< 0.001*	5.4 (2.0)	< 0.001**
No. of teeth, mean (SD)	27.3 (1.3)	26.2 (4.4)	> 0.05	23.8 (4.2)	< 0.001**
Mean (SD), % of sites with					
PPD ≥ 4 mm BoP+	5.8 (7.5)	61.6 (33.7)	< 0.001*	17.4 (12.6)	< 0.001**
PPD ≥ 6 mm	0	23.1 (18.3)	< 0.001*	19.7 (15.0)	< 0.05**
Periodontitis stage (%)					
Stage III, *n* (%)	/	15 (60.0)			
Stage IV, *n* (%)	/	10 (40.0)			

Abbreviations: BMI, body mass index; BOP, Bleeding on Probing; CAL, Clinical Attachment Level; FMBS, full‐mouth bleeding score; FMPS, full‐mouth plaque score; PPD, Probing Pocket Depth; SD, standard deviation.

* Significant differences between periodontitis patients at baseline and healthy controls.

** Significant differences between periodontitis patients pre‐ and post‐treatment.

### 3.2 Oral and Gut Metagenomics Profiles

Shotgun metagenomic sequencing generated a median of 16.2 million high‐quality non‐host reads per saliva sample and 19.4 million reads per stool sample. A total of 678 microbial species were identified across all samples. Analysis of within‐sample (alpha) diversity using the Shannon index revealed a significant increase in salivary diversity following periodontal therapy (*p* = 0.003), whereas no significant change was observed in stool samples (*p* = 0.332). Compared to healthy controls, periodontitis patients exhibited a non‐significant trend toward higher salivary diversity at baseline (ΔShannon = +0.07, *p* = 0.4), followed by a decrease post‐treatment (ΔShannon = −0.10, *p* = 0.2). In stool, both pre‐treatment and post‐treatment samples showed a non‐significant reduction in diversity relative to controls (ΔShannon = −0.02, *p* = 0.869 and ΔShannon = −0.10, *p* = 0.375, respectively). Between‐sample (beta) diversity assessed via Bray–Curtis distances showed significant compositional differences in saliva between healthy controls and periodontitis patients at baseline (PERMANOVA, *p* = 0.01), but these differences were not significant post‐treatment (*p* = 0.18). A similar trend was observed in stool, with significant divergence between controls and periodontitis samples at baseline (*p* = 0.013), but no significant difference after treatment (*p* = 0.10).

**FIGURE 1:**
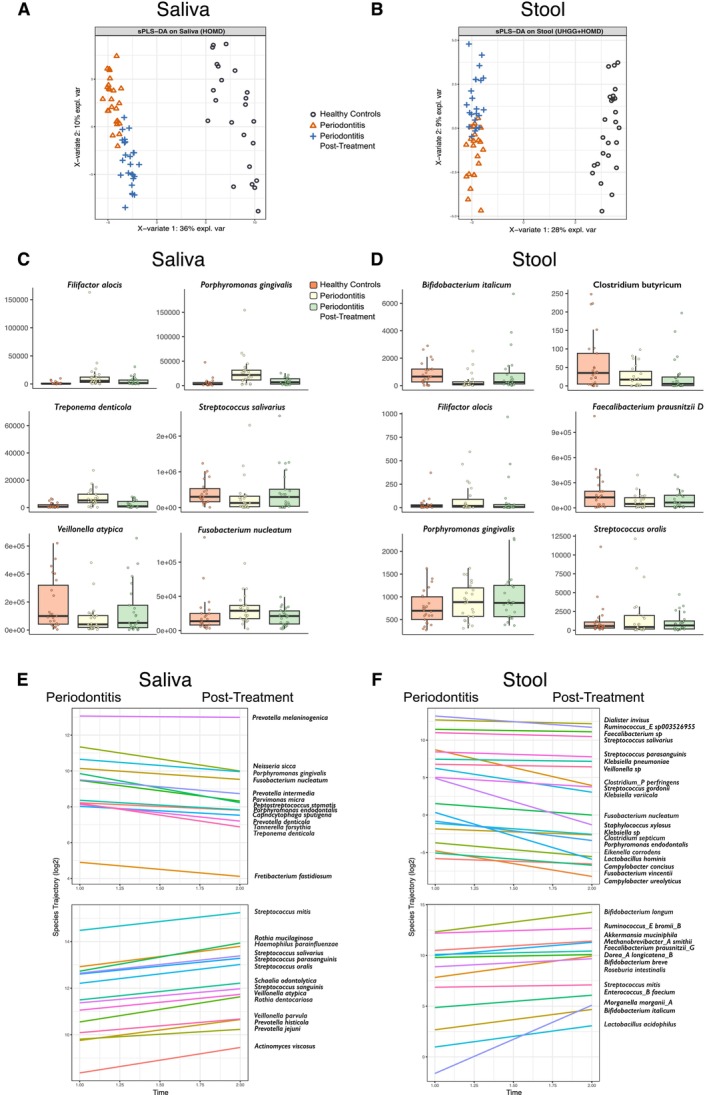
Impact of periodontitis and periodontal therapy on microbial composition across the oral–gut axis. (A–B) Supervised multivariate analysis (sPLS‐DA) of species‐level profiles revealed clear separation of healthy controls, periodontitis patients, and post‐treatment samples in saliva (A) and stool (B). (C–D) Boxplots illustrate MaAsLin2‐determined differential abundance of representative oral taxa in saliva (C) and gut taxa in stool (D), highlighting the enrichment of oral bacteria and depletion of SCFA‐producing species in periodontitis, with partial restoration following therapy. (E–F) Trajectory plots from timeOmics analysis of key species dynamics in saliva (E) and stool (F), showing significant declines in oral pathobionts and an increase of health‐associated commensals post‐therapy.

sPLS‐DA of species‐level profiles demonstrated clear separation between healthy controls, periodontitis patients, and post‐treatment samples in both saliva (Figure 1A) and stool (Figure 1B). In saliva, 246 species were significantly different in the periodontitis subjects at T0 compared to controls (*q* < 0.1 FDR; Table S2 and Figure S1). Salivary microbiota of patients at baseline showed a profile enriched with classic periodontitis‐associated species, including *Porphyromonas gingivalis* , *Tannerella forsythia* , *Treponema denticola* , *Filifactor alocis* , *Fusobacterium nucleatum* , and reduced levels of health‐associated taxa such as *Haemophilus parainfluenzae* , *Rothia mucilaginosa* , *Streptococcus salivarius* , and *Veillonella atypica* (Figure 1C). In stool, 1465 species were significantly different in the periodontitis subjects at T0 compared to controls (*q* < 0.1 FDR; Table S3 and Figure S2). Among others, the stool microbiome of periodontitis patients exhibited decreased abundance of gut commensal taxa, including *Bifidobacterium bifidum* , *B. italicum*, *Acetatifactor sp003447295*, *Faecalibacterium prausnitzii* , *Clostridium butyricum* and *Lactobacillus porci*, along with increased oral‐derived species such as *Fusobacterium nucleatum* , *Streptococcus oralis* , *P. gingivalis* and *F. alocis* (Figure 1D). Post‐therapy, partial recovery of commensals and reduction of pathogens were observed in both environments (Figure 1C,D).

Trajectory analysis of longitudinal species changes further highlighted dynamic shifts induced by periodontal therapy (Figure 1E,F). In saliva, 192 species significantly decreased after treatment (*q* < 0.1 FDR), mostly periodontitis‐associated species including *P. gingivalis* , *F. nucleatum* , *T. forsythia* , *P. intermedia* , and *T. denticola* . Conversely, 221 taxa significantly increased after therapy, including health‐associated taxa such as *Streptococcus mitis* , *S. oralis* , *Rothia mucilaginosa* , *Veillonella parvula* , *Actinomyces viscosus* , *and Neisseria* spp. (Figure 1E). In stool, putative pathogens such as *Clostridium perfringens* , *Campylobacter concisus* , and *Klebsiella pneumonia*, as well as oral taxa such as *E. corrodens* , *F. nucleatum* , and *P. endodontalis* consistently decreased post‐treatment (*q* < 0.1 FDR), while several SCFA‐producing species such as *Akkermansia muciniphila* and *Bifidobacterium breve* showed an increase (Figure 1F).

### 3.3 Oral–Gut Bacterial Leakage

**FIGURE 2:**
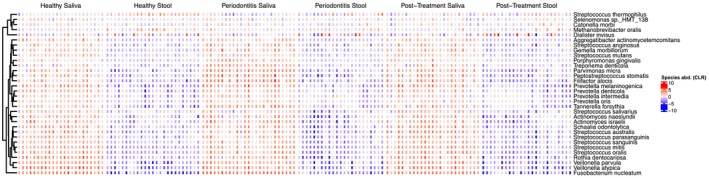
Heatmap of selected core oral species abundance across sample types. CLR‐transformed relative abundance of selected species across healthy, periodontitis, and post‐treatment saliva and stool samples. Canonical periodontal pathogens ( *P. gingivalis* , *T. denticola* ) are enriched in periodontitis saliva and reduced post‐treatment. Health‐associated commensals dominate healthy saliva and partially re‐emerge after therapy. Several oral species appear in stool samples from periodontitis patients, including *F. nucleatum* , suggesting potential oral–gut microbial transmission.

Figure 2 displays the CLR‐transformed abundance of the most representative oral bacterial species across salivary and stool specimens from the study groups. The heatmap highlights a variable presence of oral‐associated taxa in stool samples, with group‐dependent differences in relative abundance. Several species, including *F. nucleatum* , *T. denticola* , *P. intermedia* , *P. micra* , *P. stomatis* , and *T. forsythia* , were detected in both saliva and stool samples across clinical groups, with higher abundances in saliva. In stool, these species were more frequently observed in periodontitis samples, both at baseline and post‐treatment, and were minimally represented in healthy controls. Health‐associated taxa such as *S. mitis* , *S. sanguinis* , *R. dentocariosa* , and *V. parvula* were more abundant in the saliva and stool of healthy individuals, while their levels decreased variably across the other groups.

### 3.4 Functional Pathways

2892 genes were assigned using the KEGG Orthology database with HUMAnN3. Separating out each site, the control samples in saliva had a greater number of observed genes at 2165 than the periodontitis samples, while the healthy stool samples were within the range of pre‐ and post‐treatment at 2331 genes. Counts were similar within each site before and after treatment, but stool samples exhibited more observed genes overall. In total the following gene counts were observed: saliva pre‐treatment: 1947; saliva post‐treatment: 1927; stool pre‐treatment: 2330; and stool post‐treatment: 2360. As a measure of environmental consistency, we also determined the set of core genes which we defined as those present in at least 60% of samples. Saliva exhibited a higher ratio of core to observed genes at 63% in health compared to 55% in healthy stool. Disease samples followed a similar trend between sites; however, stool samples were nearly identical in ratio to health (54% pre‐ and post‐treatment) compared to 68% and 69% in saliva. Thus, while stool exhibited more genes overall, those genes were less consistently present across samples than saliva.

**FIGURE 3:**
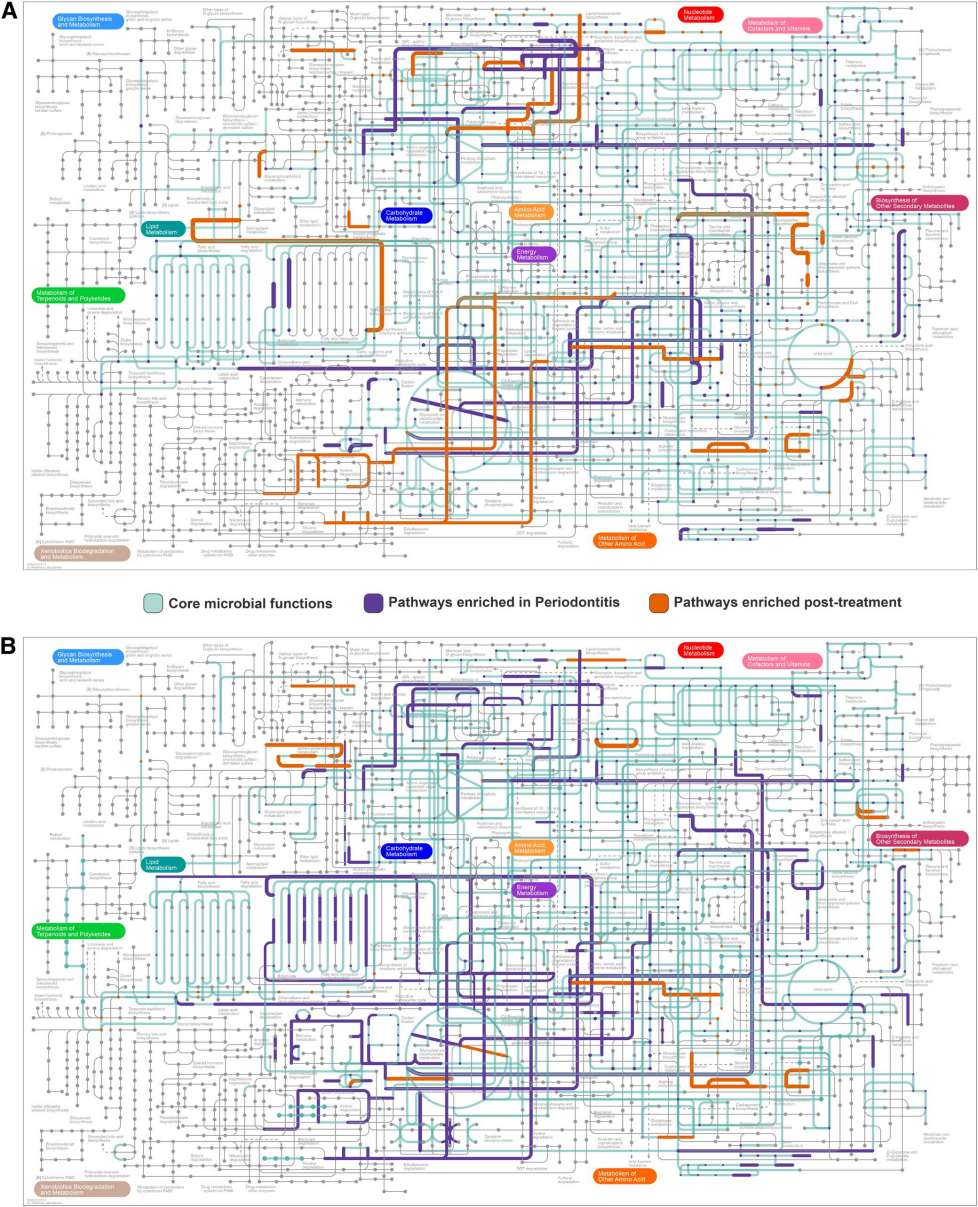
Integrated KEGG pathway maps show microbiome‐associated functional shifts in saliva (A) and stool (B) before and after Steps I–II of periodontal therapy. Green lines indicate core functions enriched in healthy controls, purple lines pathways elevated in periodontitis, and orange lines functions increased post‐treatment. In health, both niches exhibited conserved metabolic functions—central carbon metabolism, amino acid, nucleotide, and lipid metabolism, and vitamin/cofactor biosynthesis—along with glycan biosynthesis and oxidative phosphorylation in saliva and SCFA/bile acid metabolism in stool. Periodontitis was marked by enrichment of lipopolysaccharide biosynthesis, peptidoglycan turnover, amino sugar metabolism, xenobiotic degradation, motility, and drug resistance. Post‐treatment samples showed increased glycan degradation, vitamin synthesis, and SCFA/bile acid pathways, indicating partial metabolic restoration. Line thickness corresponds to the relative effect size of pathway‐level differences. All visualized pathways were derived from the genes identified being significantly different between pre‐ and post‐ using timeOmics.

Moving from counts to pathways, Figure 3 as well as Figures S3 and S4 visualizes the functional potential of microbiomes in both saliva (A) and stool (B), as inferred from KEGG pathway analysis. In both habitats, healthy controls exhibited a consistent core of microbial functions related to central carbon metabolism (glycolysis, TCA cycle, pentose phosphate pathway), amino acid metabolism (e.g., glutamate, lysine, valine), nucleotide metabolism, lipid biosynthesis, and vitamin/cofactor production (riboflavin, biotin, thiamine). In saliva, these were complemented by glycan biosynthesis and oxidative phosphorylation, while stool samples also featured short‐chain fatty acid metabolism (butanoate, propanoate) and bile acid transformation.

In both niches, periodontitis at baseline was associated with increased prevalence of pathways related to lipopolysaccharide biosynthesis, amino sugar and peptidoglycan metabolism, xenobiotic degradation, and microbial motility/defense, including flagellar assembly and drug resistance modules. These functions were more pronounced in stool, where additional enrichment was observed in pathways related to secondary metabolite biosynthesis.

Post‐treatment samples from both saliva and stool showed a distinct shift, with increased abundance of pathways involved in glycan degradation, vitamin metabolism, and SCFA and bile acid‐associated functions, indicating a reconfiguration of microbial metabolic activity following therapy.

### 3.5 Oral and Gut Metabolomics Profiles

**FIGURE 4:**
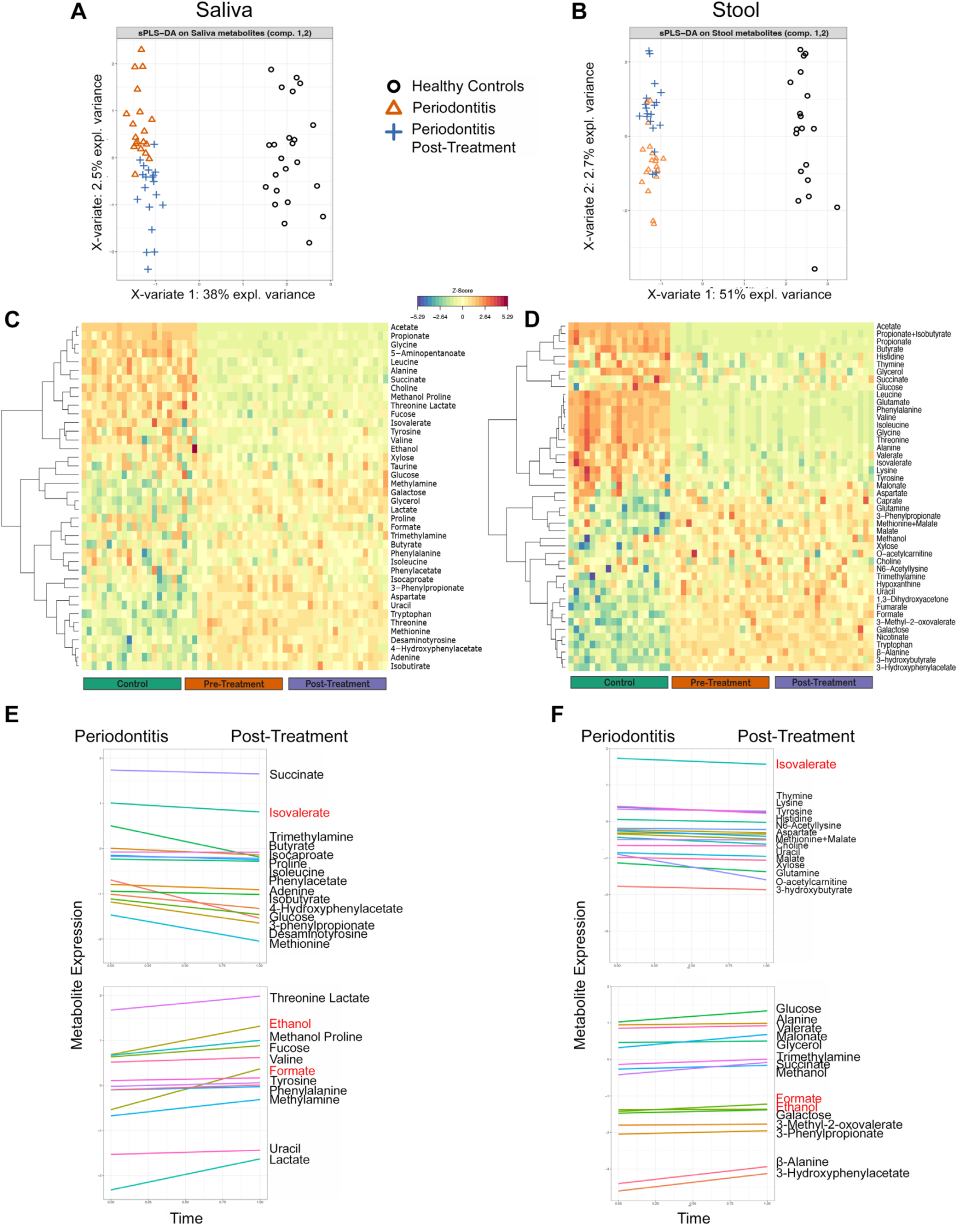
Impact of periodontitis and periodontal therapy on oral and gut metabolomic profiles. (A–B) Supervised multivariate analysis (sPLS‐DA) of NMR‐derived metabolomic profiles revealed distinct clustering of healthy controls, periodontitis patients, and post‐treatment samples in saliva (A) and stool (B), demonstrating broad metabolic shifts associated with disease and partial normalization following therapy. (C–D) Heatmaps of salivary (C) and stool (D) metabolite profiles, showing depletion of short‐chain fatty acids (SCFAs), amino acids, and sugars, and elevation of dysbiosis‐associated metabolites (trimethylamine, formate) in periodontitis patients. Post‐treatment samples exhibited moderate changes in saliva and stool. (E–F) Salivary trajectories (E) showed post‐treatment decreases in SCFAs (isolvalerate, isocaproate), amino acids (methionine), organic acids (3 − phenylpropionate), and sugars (glucose), along with increases in sugars (fucose), alcohols (ethanol, methanol), organic acids (threonine lactate, formate), and nitrogenous/nucleotide compounds (methylamine). Stool trajectories (F) revealed post‐treatment reductions in isolvalerate, tyrosine, glutamine, and O‐acetylcarnitine, and increases in glucose, malonate, succinate, formate, 3‐hydroxyphenylacetate, and β‐alanine, and indicating niche‐specific metabolic reconfiguration following therapy.

Supervised multivariate analysis of NMR‐derived metabolomic data from stool and saliva revealed distinct group‐level separation (Figure 4A,B), serving as an overall index of between‐sample dissimilarity. sPLS‐DA plots indeed demonstrated a clear separation between periodontally healthy controls, baseline periodontitis patients, and post‐treatment samples. In both samples, the metabolome of periodontitis patients at baseline was markedly distinct from controls. Post‐treatment samples showed a directional shift toward the healthy cluster, with greater convergence observed in saliva than in stool.

Heatmap analysis of single salivary metabolites (Figure 4C) revealed substantial shifts in key metabolite classes across clinical groups. In healthy controls, saliva was enriched in SCFAs (acetate, propionate, isovalerate), amino acids (valine, leucine, alanine, tyrosine, glycine, proline), and sugars (xylose, fucose), along with choline. In periodontitis, these metabolites were downregulated, while markers of dysbiosis and inflammation such as trimethylamine, methylamine, formate, lactate, and phenylacetate were elevated. Post‐treatment, slight increases in pathways involving carbohydrate metabolism (glucose, galactose, and glycerol) and amino acid metabolism (taurine) were observed, although these did not reach statistical significance.

The stool metabolomic heatmap (Figure 4D) showed consistent group‐level patterns, with pronounced differences between controls and disease. Stool samples from periodontitis patients exhibited reduced levels of SCFAs (acetate, propionate, butyrate, isovalerate, valerate), amino acids (valine, leucine, isoleucine, threonine, alanine, glycine, glutamate, phenylalanine, tyrosine), and sugars (glucose). Conversely, several inflammatory and dysbiosis‐associated metabolites, including succinate, trimethylamine, formate, and 3‐hydroxyphenylacetate, were elevated. In the heatmap visualization, post‐treatment stool samples retained a metabolic profile that remained largely distinct from healthy controls, despite underlying changes in individual metabolites.

Trajectory modeling using linear mixed models (lmms, timeOmics) was applied to salivary and fecal NMR metabolomic data to evaluate pre‐ and post‐treatment shifts in specific metabolite expression (Figure 4E,F). In saliva, post‐treatment decreases were observed in SCFAs (isovalerate, isocaproate), amino acids (methionine), organic acids (3‐phenylpropionate), and sugars (glucose). In contrast, there were increases in sugars (fucose), alcohols (ethanol, methanol), organic acids (threonine‐lactate, formate), and nitrogenous compounds (methylamine, uracil), indicating a complex metabolic shift that included both beneficial and dysbiosis‐associated metabolites. In stool, post‐treatment reductions were seen in isovalerate, tyrosine, glutamine, and O‐acetylcarnitine, while increases occurred in glucose, malonate, succinate, formate, 3‐hydroxyphenylacetate, and β‐alanine.

### 3.6 Multi‐Omics Integration

**FIGURE 5:**
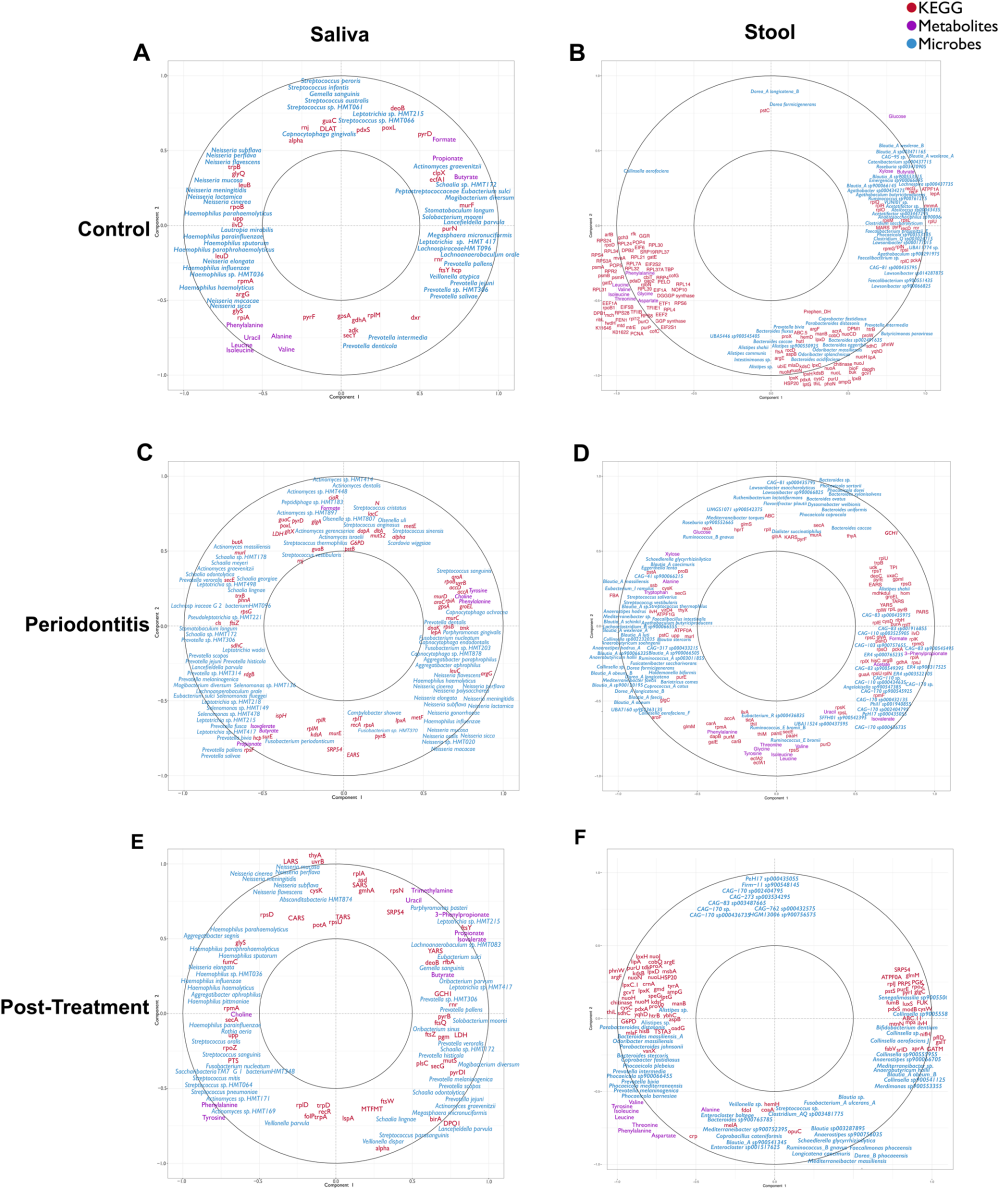
Integrated multi‐block PLS multi‐omics networks linking microbiota, functional genes, and metabolites across the oral–gut axis in health, periodontitis, and post‐treatment states. Correlation circle plots generated using mixOmics show co‐variation among microbial taxa (blue), KEGG functional orthologs (red), and metabolites (purple) in saliva (A, C, E) and stool (B, D, F) across clinical groups.

Multi‐block integration using DIABLO enabled joint analysis of salivary and stool microbiota, functional genes (KEGG pathways), and metabolites across clinical groups. Correlation circle plots (Figure 5) revealed distinct, condition‐specific networks of co‐varying features, highlighting how ecological and metabolic configurations differ in health, disease, and post‐treatment states.

In saliva of healthy controls, coherent networks linking health‐associated bacterial taxa (e.g., *Streptococcus* spp., *Gemella sanguinis* , *Actinomyces graevenitzii* ) with microbial metabolites such as SCFA (formate, butyrate), and bacterial KEGG modules including clpX, poxL, and deoB were revealed. Additionally, *Neisseria* and *Haemophilus* species co‐clustered with branched‐chain amino acids (valine, leucine, isoleucine) and biosynthetic modules such as argG (bacterial arginine biosynthesis), glyS (glycine–tRNA ligase), and pyrF (pyrimidine biosynthesis), indicating anabolic activity and microbial support for nutrient cycling.

In periodontitis at baseline, this configuration was markedly altered. Along with an increase in system complexity, disease‐associated species such as *F. nucleatum* and *P. gingivalis* became dominant, clustering with inflammatory and proteolytic metabolites, including tyrosine and phenylalanine. These taxa were also associated with bacterial functional modules such as groEL, lepA, dnaK, and rpsB, which encode molecular chaperones and ribosomal proteins involved in stress adaptation, protein folding, and translational efficiency. Collectively, this configuration reflects a shift toward a dysbiotic, metabolically hyperactive community characterized by stress response activation, proteolytic metabolism, and enhanced oxidative tolerance.

Following periodontal therapy, salivary multi‐omic networks showed partial reversion toward a health‐associated configuration. Notably, *P. gingivalis* was no longer detected in the integrative panel, while commensal taxa such as *V. parvula* , *S. mitis* , and *S. oralis* re‐emerged as central nodes. These were correlated with bacterial KEGG modules including upp (uracil salvage), secA (protein translocation), PTS (phosphotransferase system), and folP (folate biosynthesis). Together, these features suggest a trend toward nutrient recycling, as well as restoration of biosynthetic and transport functions typical of a healthy oral ecosystem.

In stool samples from periodontally healthy individuals, dominant commensals such as *Blautia*, *Roseburia*, *Faecalibacterium*, *Agathobacter*, and *Coprobacter* clustered with ribosomal protein genes (rpl, rps, rpm), ATP synthase, and glucose metabolism pathways, indicating a metabolically active and energy‐efficient community. *Bacteroides*, *Alistipes*, *Prevotella*, and *Parabacteroides* were associated with oxidative phosphorylation and amino acid metabolism (argE, hutI, purU), while *Dorea* and *Collinsella* correlated with phosphate transport (pstC) and glucose utilization. Conversely, core biosynthetic functions (i.e., ribosomal assembly, elongation factors, and amino acid biosynthesis) loaded negatively on the same axis, reflecting complementary metabolic profiles within a balanced ecosystem.

In periodontitis, the ordination revealed broader dispersion of variables, indicating increased heterogeneity in microbial–functional relationships. *Bacteroides*, *Phocaeicola*, *Lawsonibacter*, *Dysosmobacter*, and *Flavonifractor* clustered with genes for nucleotide and amino acid metabolism (thyA, pyrF) and ribosomal proteins (rplU, rpsG), consistent with intensified biosynthetic activity. In contrast, *Blautia*, *Collinsella*, *Dorea*, and *Ruminococcus* grouped with carbohydrate metabolism genes (glgC, galE, carA) and glycolysis/gluconeogenesis (FBA), suggesting enhanced fermentative metabolism within a dysbiotic network. After treatment, gut networks showed partial restoration of stability and realignment toward the healthy pattern. However, *Bacteroides*, *Parabacteroides*, and *Alistipes* remained linked to oxidative phosphorylation and lipopolysaccharide‐related functions (lpxH, lpxD, msbA), indicating residual metabolic stress and incomplete normalization of the gut ecosystem.

## 4 Discussion

This study presents the first multi‐omics characterization of the oral–gut axis in periodontitis, integrating high‐throughput whole metagenome sequencing with NMR‐based metabolomics across saliva and stool, and incorporating longitudinal follow‐up after non‐surgical periodontal therapy. Despite the known compartmentalization of the oral and gut microbiomes, a subset of bacterial species was found to be shared between saliva and stool, providing human evidence of potential microbial exchange or transit along the enteral axis. Distinct and coordinated shifts in microbial communities and their metabolic activity were identified in both environments, transitioning from periodontal health to disease, thereby highlighting the broader ecological impact of periodontitis beyond the oral cavity. Periodontal therapy up to Step II induced measurable shifts in the dysbiotic state, with directional changes observed not only in the oral microbiome but also in the gut, suggesting a systemic ecological response to local periodontal treatment.

At the taxonomic level, periodontitis was associated with an enrichment of anaerobic and proteolytic pathobionts in saliva, including *P. gingivalis* , *T. forsythia* , *T. denticola* , *F. alocis* , *F. nucleatum* , and reduced levels of health‐associated taxa such as *H. parainfluenzae* , *R. mucilaginosa* , *Streptococcus* spp., and *Veillonella* spp. These findings are consistent with classical studies linking specific microbial complexes to disease, as well as recent metagenomic analyses showing community‐wide alterations in oral dysbiosis [7, 64]. In stool, dysbiotic trends were also observed, including increased prevalence of oral‐origin taxa such as *F. nucleatum* , *S. oralis* , *P. gingivalis* and *F. alocis* , as well as reduced counts of symbiotic taxa such as *Bifidobacterium* spp., *F. prausnitzii* , and *Lactobacillus* spp. These results support the hypothesis that periodontitis may influence the distal gut microbial ecosystems through enteral introgression of microorganisms or their genetic material, systemic inflammatory signaling, or mucosal barrier disruption [21, 28].

Initial periodontal therapy resulted in a partial reversion of these alterations. Several commensal taxa re‐emerged in saliva, and stool communities displayed less consistent but directionally similar changes. Interestingly, oral taxa such as *E. corrodens* , *F. nucleatum* , and *P. endodontalis* declined sharply in fecal samples post‐treatment, mirroring reductions in the oral cavity. These observations align with previous research highlighting the short‐term microbial effects of non‐surgical periodontal treatment [31, 32] while adding taxonomic depth, and suggest that therapy targeting the oral niche may exert an indirect influence on gut microbial composition [23]. It should be noted, however, that the present study evaluated responses only to Steps I–II of therapy, which typically do not achieve full clinical resolution, and that the follow‐up period was limited to 3 months [46, 65].

In terms of oral and gut similarity, a subset of species was shared between saliva and stool in the majority of subjects [29, 66, 67]. Although most core taxa remained habitat‐specific, several oral commensals—including *S. mitis* , *S. sanguinis* , *R. dentocariosa* , and *V. parvula* —were consistently detected in both saliva and stool across all clinical groups, representing a stable cross‐habitat microbiome. The overall degree of species overlap remained relatively constant across groups; however, a slight increase in the oral–gut translocation of pathobionts was observed in individuals with periodontitis [30, 32]. Notably, this study reports for the first time the concurrent detection of key oral pathobionts at the species level—including *F. nucleatum* , *T. denticola* , *P. intermedia* , *P. micra* , *P. stomatis* , and *T. forsythia—*in both saliva and stool, primarily in systemically healthy patients with periodontitis. This observation raises the hypothesis that inflammatory conditions may promote the persistence or selective enrichment of specific organisms across anatomical compartments [19, 23, 24]. Compared to previous investigations [68, 69, 70], the enhanced taxonomic resolution achieved in this study was likely due to the use of whole genome shotgun sequencing and the integration of niche‐specific databases (HOMD for oral samples and UHGG for gut samples), which extremized the sensitivity for detecting oral species in the gut. It is also noteworthy that abnormal colonization of the gut by oral bacteria has been reported in individuals with achlorhydria or PPI use [71], yet in the present study all participants were systemically healthy and not under such medications, minimizing this potential confounding factor.

Functional profiling of microbial gene content provided deeper insight into the ecological consequences of periodontitis. Both saliva and stool samples from affected individuals were enriched in pathways related to lipopolysaccharide biosynthesis, flagellar assembly, and xenobiotic degradation—features indicative of a pro‐inflammatory, environmentally resilient microbiota [72, 73, 74]. These pro‐inflammatory and dysbiosis‐associated signatures parallel those observed in mucosal inflammatory conditions such as IBD [24, 75, 76], and colorectal cancer [77], and may contribute to low‐grade systemic inflammation and metabolic dysfunction [21]. Following therapy, functional recovery was not complete in both oral and gut niches. However, an increased representation of homeostasis‐related functions was observed, including vitamin biosynthesis (e.g., riboflavin, biotin), SCFA metabolism, and glycan degradation, particularly in the oral environment. These findings suggest a metabolic rebalancing of the microbiome toward a more host‐compatible state, potentially contributing to reduced inflammatory burden.

Metabolomic profiling provided an integrated perspective on how microbial and host interactions translate into systemic biochemical changes across the oral–gut axis. Microorganisms release hundreds of proteins and peptides that can be metabolized into a range of bioactive compounds, many of which exhibit cytotoxic or immunomodulatory properties [78]. These microbial metabolites may partly account for the capacity of oral–gut microbial transmission to alter the intestinal microbiota and potentially influence disease pathogenesis. Once in the gut, oral taxa can indeed engage in diverse immunological and inflammatory pathways. Experimental models involving oral microbiota or saliva transfer have demonstrated altered intestinal expression of cytokines, chemokines, and tight junction proteins, supporting a role in promoting mucosal inflammation and increased intestinal permeability [79, 80]. NMR metabolomic data corroborated the microbial findings, revealing clear biochemical signatures distinguishing periodontal health from disease [81, 82]. In both saliva and stool, periodontitis was indeed associated with marked reductions in SCFAs, including acetate, propionate, butyrate, isovalerate, and valerate, molecules that are known to support gut epithelial barrier function, modulate host immunity, and exert anti‐inflammatory effects [83, 84]. Amino acids and carbohydrate metabolites such as glucose, xylose, and galactose were also consistently depleted, while dysbiosis‐associated metabolites including succinate, trimethylamine, methylamine, formate, and 3‐hydroxyphenylacetate were elevated, particularly in stool samples. These shifts mirror metabolic patterns reported in IBD, metabolic syndrome, and cardiovascular diseases, providing further mechanistic linkage between periodontitis‐induced oral dysbiosis and systemic inflammatory risk [85, 86]. Importantly, while periodontal therapy led to clear taxonomic and partial functional recovery, metabolomic normalization was slower and more incomplete. This temporal lag is consistent with prior studies showing that mucosal metabolomes, being the integrated product of both microbiota and host factors, tend to be highly resilient [66, 75]. Together, these findings highlight that rebalancing systemic metabolic networks after local periodontal therapy may require sustained intervention and longer time frames than microbial shifts alone might suggest.

Multi‐omics approaches are highly recommended in research; albeit their implementation is still at its infancy, especially in saliva [87, 88]. In periodontal health, commensal organisms such as *S. mitis* , *G. sanguinis* , and *A. graevenitzii* clustered with SCFA and biosynthetic KEGG pathways, suggesting a functionally stable ecosystem supporting mucosal homeostasis. By contrast, in periodontitis, hallmark pathogens like *P. gingivalis* and *F. nucleatum* were associated with stress‐related chaperone proteins and metabolites linked to proteolysis and aromatic amino acid degradation, such as tyrosine and phenylalanine. These disease‐associated configurations reflected a microbial community primed for inflammation and tissue breakdown [75]. Following therapy, commensal species and functional modules linked to nutrient recycling and transport re‐emerged, indicating a shift toward metabolic rebalancing, though not full resolution. Compared to saliva, gut networks were more complex and less responsive to therapy within the study timeframe, likely reflecting both the higher ecological diversity of the intestinal microbiota and the indirect nature of gut modulation [89, 90]. These findings underscore the value of integrative, cross‐niche approaches to capture ecological and functional dynamics during periodontal disease and recovery. Future mechanistic studies are needed to dissect how oral inflammation and microbial translocation shape the gut ecosystem across time.

While these results support the view that periodontal inflammation and dysbiosis perturb both local and distal microbial environments, the possibility of a bidirectional effect must be considered [91, 92]. Alterations in gut microbial composition and function could also influence periodontal homeostasis via immunological priming, nutrient metabolism, or systemic immune modulation [93]. The observed responsiveness of the gut microbiome to oral therapy indicates a contribution of the downstream effect, but longitudinal intervention studies and mechanistic work are needed to clarify the co‐existence of also an upstream directionality in the oral–gut axis.

The present study offers a robust framework for investigating causal links between oral dysbiosis and gut ecosystem disruption through a multi‐omics lens. Building on these findings, some limitations and avenues for future research can be identified. Although shotgun metagenomics enables the detection of all microbial domains, the present analysis focused on bacterial taxa and non‐bacterial components, such as fungi and archaea, were not systematically characterized. Additionally, while subspecies‐level resolution (e.g., for *F. nucleatum* ) was attainable, the study did not aim to analyze within‐species variation in depth. While shotgun metagenomics offered detailed taxonomic and functional insights, future studies could benefit from complementary techniques, such as targeted culturing and metaproteomics, to more directly assess microbial viability and activity, particularly for oral‐derived pathobionts identified in stool. Metatranscriptomics may also offer additional insight but remains technically challenging in fecal samples due to RNA instability. Another limitation concerns the longitudinal design. Healthy controls were sampled at a single timepoint, precluding intra‐individual temporal comparisons. Moreover, although the 3‐month post‐treatment follow‐up was sufficient to capture early microbial and metabolic shifts, a longer observation period may be required to assess the stability and functional resilience of the oral and gut microbiomes [94]. Furthermore, saliva was selected as the oral sampling medium given the study's objective of evaluating potential translocation of oral taxa to the gut via the enteral route. However, its use may underrepresent niche‐specific commensals and pathobionts compared with subgingival plaque. Finally, although the study cohort was carefully controlled and free from major confounding factors (e.g., recent antibiotic use, PPI therapy, unbalanced diet) [95], larger and more diverse populations, coupled with integrated host immune profiling (i.e., miRNA) [96] and assessment of epithelial barrier integrity, will be critical to fully elucidate the systemic consequences of modulating the oral–gut microbiome axis.

## 5 Conclusions

This study offers a high‐resolution ecological characterization of periodontitis across oral and gut environments in systemically healthy individuals, based on an integrated multi‐omics approach combining shotgun metagenomics and NMR‐based metabolomics. Periodontitis was associated with coordinated alterations in microbial composition, functional potential, and metabolic output in both saliva and stool, with relevant overlap of disease‐associated features across niches. Following Steps I–II of periodontal therapy, partial restoration of taxonomic and metabolic profiles was observed, more evident in the oral cavity but even detectable in the gut. These findings support the concept that periodontitis may influence distal microbial ecosystems and suggest that modulation of the oral–gut axis could represent a clinically relevant mechanism through which periodontal treatment contributes to systemic health.

## Author Contributions

Giacomo Baima contributed to conception and design, acquisition, analysis, and interpretation; drafted and critically revised the manuscript. Shareef Dabdoub contributed to data analysis and interpretation; drafted and critically revised the manuscript. Vivek Thumbigere‐Math contributed to data analysis and interpretation; drafted and critically revised the manuscript. Davide Giuseppe Ribaldone and Gian Paolo Caviglia contributed to data acquisition and drafted the manuscript. Leonardo Tenori, Linda Fantato, Alessia Vignoli contributed to metabolomics analyses and critically revised the manuscript. Mario Romandini contributed to design, data interpretation, and critically revised the manuscript. Ilario Ferrocino contributed to design, data acquisition and analysis; critically revised the manuscript. Mario Aimetti contributed to conception and design, and critically revised the manuscript. All authors gave their final approval and agree to be accountable for all aspects of the work.

## Conflicts of Interest

The authors declare no conflicts of interest.

## Supporting information


**Appendix S1:** jre70055‐sup‐0001‐Appendix.docx.

**Figure S1:** Heatmap of species that most separate the three study groups by sparse Partial Least Squares Discriminant Analysis (sPLS‐DA) in saliva. CSaliva_T0; healthy controls, PSaliva_T0, periodontitis patients at baseline; PSaliva_T1, post‐treatment samples.

**Figure S2:** Heatmap of species that most separate the three study groups by sparse Partial Least Squares Discriminant Analysis (sPLS‐DA) in stool. CStool_T0; healthy controls, PStool_T0, periodontitis patients at baseline; PStool_T1, post‐treatment samples.

**Figure S3:** KEGG pathway differences in salivary microbiomes between periodontitis patients pre‐ and post‐treatment. Dot plots illustrate differentially enriched microbial KEGG pathways in saliva samples from individuals with periodontitis pre‐ and post‐treatment. (Top) Pathways significantly increased in post‐treatment included central carbon metabolism, amino acid and nucleotide biosynthesis, oxidative phosphorylation, phosphotransferase systems (PTS), and various sugar and energy metabolism modules. (Bottom) Pathways reduced in post‐treatment encompassed glycan biosynthesis, lipopolysaccharide biosynthesis, butanoate metabolism, DNA repair and replication, and several cofactor and amino acid biosynthetic functions. Dot size corresponds to the number of annotated genes per pathway, and color represents adjusted q‐values. These data indicate that treatment is associated with distinct metabolic reprogramming of the salivary microbiome, reflecting increased energy generation and stress response functions alongside reductions in host‐compatible and biosynthetic pathways.

**Figure S4:** Functional KEGG pathway differences in stool microbiomes between periodontitis patients pre‐ and post‐treatment. Dot plots display KEGG pathway enrichment analysis of microbial genes differentially abundant in stool samples from periodontitis patients pre‐ and post‐treatment. (Top) Pathways significantly enriched in post‐treatment were predominantly related to central carbon metabolism, amino acid and nucleotide metabolism, phosphotransferase systems (PTS), microbial motility (e.g., flagellar assembly, chemotaxis), and stress‐associated functions. (Bottom) Pathways decreased in post‐treatment included biosynthetic modules involved in oxidative phosphorylation, ribosome formation, biotin metabolism, glycan degradation, and multiple DNA repair and replication processes. Dot size reflects the number of genes per pathway; color denotes adjusted q‐values. These findings suggest that periodontitis treatment functionally reconfigures the gut microbiome toward energy harvesting, environmental resilience, and pro‐inflammatory potential, with depletion of host‐compatible biosynthetic and regulatory functions.

**Table S1:** jre70055‐sup‐0006‐TableS1.docx.

**Table S2:** jre70055‐sup‐0007‐TableS2.xlsx.

**Table S3:** jre70055‐sup‐0008‐TableS3.xlsx.

## Data Availability

Metagenomic sequencing data are available through the NCBI Sequence Read Archive (SRA) under BioProject SUB15720312.

## References

[1] S. Molloy , “Snapshot of a Superorganism,” Nature Reviews. Microbiology 4, no. 7 (2006): 491.

[2] J. A. Gilbert , M. J. Blaser , J. G. Caporaso , J. K. Jansson , S. V. Lynch , and R. Knight , “Current Understanding of the Human Microbiome,” Nature Medicine 24, no. 4 (2018): 392–400. 10.1038/nm.4517 PMC7043356 29634682

[3] W. G. Wade , “The Oral Microbiome in Health and Disease,” Pharmacological Research 69, no. 1 (2013): 137–143. 23201354 10.1016/j.phrs.2012.11.006

[4] G. Hajishengallis , “Periodontitis: From Microbial Immune Subversion to Systemic Inflammation,” Nature Reviews. Immunology 15, no. 1 (2015): 30–44. 10.1038/nri3785 PMC4276050 25534621

[5] P. N. Papapanou , M. Sanz , N. Buduneli , et al., “Periodontitis: Consensus Report of Workgroup 2 of the 2017 World Workshop on the Classification of Periodontal and Peri‐Implant Diseases and Conditions,” Journal of Periodontology 89, no. S1 (2018): S173–S182. 29926951 10.1002/JPER.17-0721

[6] G. G. Nascimento , S. Alves‐Costa , and M. Romandini , “Burden of Severe Periodontitis and Edentulism in 2021, With Projections up to 2050: The Global Burden of Disease 2021 Study,” Journal of Periodontal Research 59 (2024): 823–867. 39192495 10.1111/jre.13337

[7] A. Antezack , D. Etchecopar‐Etchart , B. La Scola , and V. Monnet‐Corti , “New Putative Periodontopathogens and Periodontal Health‐Associated Species: A Systematic Review and Meta‐Analysis,” Journal of Periodontal Research 58, no. 5 (2023): 893–906. 37572051 10.1111/jre.13173

[8] G. Baima , M. Arce , M. Romandini , and T. Van Dyke , “Inflammatory and Immunological Basis of Periodontal Diseases,” Journal of Periodontal Research (2025). 10.1111/jre.70040 41065279

[9] I. Darby , “Risk Factors for Periodontitis & Peri‐Implantitis,” Periodontology 2000 90, no. 1 (2022): 9–12. 35913624 10.1111/prd.12447 PMC9804916

[10] C. Marruganti , H. S. Shin , S. J. Sim , S. Grandini , A. Laforí , and M. Romandini , “Air Pollution as a Risk Indicator for Periodontitis,” Biomedicine 11, no. 2 (2023): 443. 10.3390/biomedicines11020443 PMC9953183 36830979

[11] C. Marruganti , M. Romandini , C. Gaeta , et al., “Healthy Lifestyles Are Associated With a Better Response to Periodontal Therapy: A Prospective Cohort Study,” Journal of Clinical Periodontology 50 (2023): 1089–1100. 37013691 10.1111/jcpe.13813

[12] G. Hajishengallis and R. J. Lamont , “Beyond the Red Complex and Into More Complexity: The Polymicrobial Synergy and Dysbiosis (PSD) Model of Periodontal Disease Etiology,” Molecular Oral Microbiology 27, no. 6 (2012): 409–419. 23134607 10.1111/j.2041-1014.2012.00663.x PMC3653317

[13] G. Hajishengallis and T. Chavakis , “Local and Systemic Mechanisms Linking Periodontal Disease and Inflammatory Comorbidities,” Nature Reviews. Immunology 21, no. 7 (2021): 426–440. 10.1038/s41577-020-00488-6 PMC7841384 33510490

[14] G. Baima , M. Minoli , D. S. Michaud , et al., “Periodontitis and Risk of Cancer: Mechanistic Evidence,” Periodontology 2000 96, no. 1 (2024): 83–94. 38102837 10.1111/prd.12540 PMC11579815

[15] C. Marruganti , M. Romandini , C. Gaeta , et al., “Treatment of Periodontitis Ameliorates the Severity and Extent of Psoriasis—A Randomized Clinical Trial,” Journal of Periodontal Research 60 (2024): 134–143. 38899599 10.1111/jre.13314

[16] G. N. Antonoglou , M. Romandini , J. H. Meurman , M. Surakka , S. J. Janket , and M. Sanz , “Periodontitis and Edentulism as Risk Indicators for Mortality: Results From a Prospective Cohort Study With 20 Years of Follow‐Up,” Journal of Periodontal Research 58, no. 1 (2023): 12–21. 36282792 10.1111/jre.13061 PMC10092146

[17] M. Wang , Z. Wang , D. Zhao , Y. Yu , and F. Wei , “Periodontitis Causally Affects the Brain Cortical Structure: A Mendelian Randomization Study,” Journal of Periodontal Research 59, no. 2 (2024): 381–386. 38059384 10.1111/jre.13222

[18] J. Xiang , J. Cao , J. Shen , et al., “Bioinformatics Analysis Reveals the Potential Common Genes and Immune Characteristics Between Atrial Fibrillation and Periodontitis,” Journal of Periodontal Research 59, no. 1 (2024): 104–118. 37971162 10.1111/jre.13192

[19] S. Kitamoto , H. Nagao‐Kitamoto , Y. Jiao , et al., “The Intermucosal Connection Between the Mouth and Gut in Commensal Pathobiont‐Driven Colitis,” Cell 182, no. 2 (2020): 447–462.e14. 32758418 10.1016/j.cell.2020.05.048 PMC7414097

[20] K. Yang , Z. Zhang , Q. Zhang , et al., “Potential Diagnostic Markers and Therapeutic Targets for Periodontitis and Alzheimer's Disease Based on Bioinformatics Analysis,” Journal of Periodontal Research 59, no. 2 (2024): 366–380. 38189472 10.1111/jre.13220

[21] S. Kitamoto and N. Kamada , “Untangling the Oral‐Gut Axis in the Pathogenesis of Intestinal Inflammation,” International Immunology 34, no. 9 (2022): 485–490. 35716367 10.1093/intimm/dxac027 PMC9447993

[22] G. Baima , D. G. Ribaldone , M. Muwalla , et al., “Can Periodontitis Affect the Health and Disease of the Digestive System? A Comprehensive Review of Epidemiological Evidence and Biological Mechanisms,” Current Oral Health Reports 8, no. 4 (2021): 96–106.

[23] G. Baima , D. G. Ribaldone , F. Romano , M. Aimetti , and M. Romandini , “The Gum‐Gut Axis: Periodontitis and the Risk of Gastrointestinal Cancers,” Cancers (Basel) 15, no. 18 (2023): 4594. 37760563 10.3390/cancers15184594 PMC10526746

[24] H. Tanwar , J. M. Gnanasekaran , D. Allison , et al., “Unravelling the Oral‐Gut Axis: Interconnection Between Periodontitis and Inflammatory Bowel Disease, Current Challenges, and Future Perspective,” Journal of Crohn's & Colitis 18, no. 8 (2024): 1319–1341. 10.1093/ecco-jcc/jjae028 PMC11324343 38417137

[25] A. L. A. da Costa , M. A. Soares , T. G. B. Lourenço , et al., “Periodontal Pathogen *Aggregatibacter actinomycetemcomitans* JP2 Correlates With Colonic Leukocytes Decrease and Gut Microbiome Imbalance in Mice,” Journal of Periodontal Research 59, no. 5 (2024): 961–973. 38757372 10.1111/jre.13288

[26] X. Wang , Z. Li , H. Zhou , Q. Liu , X. Zhang , and F. Hu , “Periodontitis Exacerbates Colorectal Cancer by Altering Gut Microbiota‐Derived Metabolomics in Mice,” Journal of Periodontal Research 60, no. 12 (2025): 1254–1264. 10.1111/jre.13380 39843386

[27] L. Tan , Y. He , T. Wang , X. Gao , W. Fan , and B. Fan , “A Mendelian Randomization Study Between Chronic Periodontitis and Non‐Alcoholic Fatty Liver Disease,” Journal of Periodontal Research 59, no. 2 (2024): 346–354. 38102730 10.1111/jre.13218

[28] K. Atarashi , W. Suda , C. Luo , et al., “Ectopic Colonization of Oral Bacteria in the Intestine Drives TH1 Cell Induction and Inflammation,” Science 358, no. 6361 (2017): 359–365. 29051379 10.1126/science.aan4526 PMC5682622

[29] T. S. Schmidt , M. R. Hayward , L. P. Coelho , et al., “Extensive Transmission of Microbes Along the Gastrointestinal Tract,” eLife 8 (2019): e42693. 30747106 10.7554/eLife.42693 PMC6424576

[30] T. G. B. Lourenço , A. M. de Oliveira , G. Tsute Chen , and A. P. V. Colombo , “Oral‐Gut Bacterial Profiles Discriminate Between Periodontal Health and Diseases,” Journal of Periodontal Research 57, no. 6 (2022): 1227–1237. 36261869 10.1111/jre.13059

[31] A. M. de Oliveira , T. G. B. Lourenço , and A. P. V. Colombo , “Impact of Systemic Probiotics as Adjuncts to Subgingival Instrumentation on the Oral‐Gut Microbiota Associated With Periodontitis: A Randomized Controlled Clinical Trial,” Journal of Periodontology 93, no. 1 (2022): 31–44. 34028826 10.1002/JPER.21-0078

[32] G. Baima , I. Ferrocino , V. Del Lupo , et al., “Effect of Periodontitis and Periodontal Therapy on Oral and Gut Microbiota,” Journal of Dental Research 103, no. 4 (2024): 359–368. 38362600 10.1177/00220345231222800

[33] A. Krajewski , J. Perussolo , P. Ercal , N. Gkranias , and N. Donos , “The Effect of Non‐Surgical Periodontal Therapy on Subgingival Microbiota: A Systematic Review and Meta‐Analysis,” Journal of Periodontal Research (2025). 10.1111/jre.13409 PMC12640219 40347039

[34] P. S. Yu , C. C. Tu , N. Wara‐Aswapati , et al., “Microbiome of Periodontitis and Peri‐Implantitis Before and After Therapy: Long‐Read 16S rRNA Gene Amplicon Sequencing,” Journal of Periodontal Research 59, no. 4 (2024): 657–668. 38718089 10.1111/jre.13269

[35] N. Segata , J. Izard , L. Waldron , et al., “Metagenomic Biomarker Discovery and Explanation,” Genome Biology 12, no. 6 (2011): R60. 21702898 10.1186/gb-2011-12-6-r60 PMC3218848

[36] C. Quince , A. W. Walker , J. T. Simpson , N. J. Loman , and N. Segata , “Shotgun Metagenomics, From Sampling to Analysis,” Nature Biotechnology 35, no. 9 (2017): 833–844. 10.1038/nbt.3935 28898207

[37] Y. Zhao , L. Song , H. Y. Li , et al., “Metagenomic Insights Into the Subgingival Microbiome in Periodontal Health and Different Grades of Periodontitis,” Journal of Periodontal Research 60 (2025): 788–798. 40344212 10.1111/jre.13408

[38] A. Alexandre , À. Gerard , I. Sergio , et al., “Geographic Influence on Subgingival Microbiota in Health and Periodontitis: A Multinational Shotgun Metagenomic Study,” Journal of Periodontal Research 60 (2025): 910–922. 40202358 10.1111/jre.13406

[39] I. Ferrocino , K. Rantsiou , R. McClure , et al., “The Need for an Integrated Multi‐OMICs Approach in Microbiome Science in the Food System,” Comprehensive Reviews in Food Science and Food Safety 22, no. 2 (2023): 1082–1103. 36636774 10.1111/1541-4337.13103

[40] Y. Zheng , Y. Liu , J. Yang , et al., “Multi‐Omics Data Integration Using Ratio‐Based Quantitative Profiling With Quartet Reference Materials,” Nature Biotechnology 42, no. 7 (2024): 1133–1149. 10.1038/s41587-023-01934-1 PMC11252085 37679543

[41] M. Feres , B. Retamal‐Valdes , C. Gonçalves , L. Cristina Figueiredo , and F. Teles , “Did Omics Change Periodontal Therapy?,” Periodontology 2000 85, no. 1 (2021): 182–209. 33226695 10.1111/prd.12358

[42] F. A. Scannapieco and A. Dongari‐Bagtzoglou , “Dysbiosis Revisited. Understanding the Role of the Oral Microbiome in the Pathogenesis of Gingivitis and Periodontitis: A Critical Assessment,” Journal of Periodontology 92, no. 8 (2021): 1071. 33902163 10.1002/JPER.21-0120 PMC8380683

[43] I. Huybrechts , R. Miglio , L. Mistura , et al., “Relative Validity of an Italian EPIC Food Frequency Questionnaire for Dietary Factors in Children and Adolescents. A Rizzoli Orthopedic Institute Study,” Nutrients 13, no. 4 (2021): 1245. 33918879 10.3390/nu13041245 PMC8069881

[44] M. Sanz , D. Herrera , M. Kebschull , et al., “Treatment of Stage I–III Periodontitis—The EFP S3 Level Clinical Practice Guideline,” Journal of Clinical Periodontology 47, no. S22 (2020): 4–60. 32383274 10.1111/jcpe.13290 PMC7891343

[45] J. Suvan , Y. Leira , F. M. Moreno Sancho , F. Graziani , J. Derks , and C. Tomasi , “Subgingival Instrumentation for Treatment of Periodontitis. A Systematic Review,” Journal of Clinical Periodontology 47, no. S22 (2020): 155–175. 31889320 10.1111/jcpe.13245

[46] F. Ferrarotti , G. Baima , M. Rendinelli , et al., “Pocket Closure After Repeated Subgingival Instrumentation: A Stress Test to the EFP Guideline for Stage III‐IV Periodontitis,” Clinical Oral Investigations 27 (2023): 6701–6708. 37773418 10.1007/s00784-023-05279-6 PMC10630226

[47] G. Baima , G. Iaderosa , M. Corana , et al., “Macro and Trace Elements Signature of Periodontitis in Saliva: A Systematic Review With Quality Assessment of Ionomics Studies,” Journal of Periodontal Research 57, no. 1 (2022): 30–40. 34837226 10.1111/jre.12956 PMC9298699

[48] B. Langmead and S. L. Salzberg , “Fast Gapped‐Read Alignment With Bowtie 2,” Nature Methods 9, no. 4 (2012): 357–359. 22388286 10.1038/nmeth.1923 PMC3322381

[49] M. Schubert , S. Lindgreen , and L. Orlando , “AdapterRemoval v2: Rapid Adapter Trimming, Identification, and Read Merging,” BMC Research Notes 9, no. 1 (2016): 88. 26868221 10.1186/s13104-016-1900-2 PMC4751634

[50] Q. Zhu , S. Huang , A. Gonzalez , et al., “Phylogeny‐Aware Analysis of Metagenome Community Ecology Based on Matched Reference Genomes While Bypassing Taxonomy,” MSystems 7, no. 2 (2022): e00167‐22. 35369727 10.1128/msystems.00167-22 PMC9040630

[51] F. Beghini , L. J. McIver , A. Blanco‐Míguez , et al., “Integrating Taxonomic, Functional, and Strain‐Level Profiling of Diverse Microbial Communities With bioBakery 3,” eLife 10 (2021): e65088. 33944776 10.7554/eLife.65088 PMC8096432

[52] V. Ghini , G. Meoni , A. Vignoli , et al., “Fingerprinting and Profiling in Metabolomics of Biosamples,” Progress in Nuclear Magnetic Resonance Spectroscopy 138–139 (2023): 105–135. 10.1016/j.pnmrs.2023.10.002 38065666

[53] A. Vignoli , V. Ghini , G. Meoni , et al., “High‐Throughput Metabolomics by 1D NMR,” Angewandte Chemie International Edition 58, no. 4 (2019): 968–994. 29999221 10.1002/anie.201804736 PMC6391965

[54] J. G. Caporaso , J. Kuczynski , J. Stombaugh , et al., “QIIME Allows Analysis of High‐Throughput Community Sequencing Data,” Nature Methods 7, no. 5 (2010): 335–336. 20383131 10.1038/nmeth.f.303 PMC3156573

[55] S. M. Dabdoub , M. L. Fellows , A. D. Paropkari , et al., “PhyloToAST: Bioinformatics Tools for Species‐Level Analysis and Visualization of Complex Microbial Datasets,” Scientific Reports 6 (2016): 29123. 27357721 10.1038/srep29123 PMC4928119

[56] H. Mallick , A. Rahnavard , L. J. McIver , et al., “Multivariable Association Discovery in Population‐Scale Meta‐Omics Studies,” PLoS Computational Biology 17, no. 11 (2021): e1009442. 34784344 10.1371/journal.pcbi.1009442 PMC8714082

[57] A. Bodein , M. P. Scott‐Boyer , O. Perin , K. A. Lê Cao , and A. Droit , “timeOmics: An R Package for Longitudinal Multi‐Omics Data Integration,” Bioinformatics 38, no. 2 (2022): 577–579. 34554215 10.1093/bioinformatics/btab664

[58] F. Rohart , B. Gautier , A. Singh , and K. A. L. Cao , “mixOmics: An R Package for ‘Omics Feature Selection and Multiple Data Integration’,” PLoS Computational Biology 13, no. 11 (2017): e1005752. 29099853 10.1371/journal.pcbi.1005752 PMC5687754

[59] B. Liquet , K. A. Lê Cao , H. Hocini , and R. Thiébaut , “A Novel Approach for Biomarker Selection and the Integration of Repeated Measures Experiments From Two Assays,” BMC Bioinformatics 13 (2012): 325. 23216942 10.1186/1471-2105-13-325 PMC3627901

[60] Y. Darzi , I. Letunic , P. Bork , and T. Yamada , “iPath3.0: Interactive Pathways Explorer v3,” Nucleic Acids Research 46, no. W1 (2018): W510–W513. 29718427 10.1093/nar/gky299 PMC6031023

[61] I. González , K. A. L. Cao , M. J. Davis , and S. Déjean , “Visualising Associations Between Paired ‘Omics’ Data Sets,” Biodata Mining 5, no. 1 (2012): 19. 23148523 10.1186/1756-0381-5-19 PMC3630015

[62] B. J. Kelly , R. Gross , K. Bittinger , et al., “Power and Sample‐Size Estimation for Microbiome Studies Using Pairwise Distances and PERMANOVA,” Bioinformatics 31, no. 15 (2015): 2461–2468. 25819674 10.1093/bioinformatics/btv183 PMC4514928

[63] F. Bhinderwala and R. Powers , “NMR Metabolomics Protocols for Drug Discovery,” Methods in Molecular Biology 2037 (2019): 265–311. 31463851 10.1007/978-1-4939-9690-2_16 PMC7025395

[64] L. Abusleme , A. K. Dupuy , N. Dutzan , et al., “The Subgingival Microbiome in Health and Periodontitis and Its Relationship With Community Biomass and Inflammation,” ISME Journal 7, no. 5 (2013): 1016–1025. 23303375 10.1038/ismej.2012.174 PMC3635234

[65] F. Citterio , G. Gualini , M. Chang , et al., “Pocket Closure and Residual Pockets After Non‐Surgical Periodontal Therapy: A Systematic Review and Meta‐Analysis,” Journal of Clinical Periodontology 49, no. 1 (2022): 2–14. 34517433 10.1111/jcpe.13547 PMC9298904

[66] The Human Microbiome Project Consortium , “Structure, Function and Diversity of the Healthy Human Microbiome,” Nature 486, no. 7402 (2012): 207–214. 22699609 10.1038/nature11234 PMC3564958

[67] T. Ding and P. D. Schloss , “Dynamics and Associations of Microbial Community Types Across the Human Body,” Nature 509, no. 7500 (2014): 357–360. 24739969 10.1038/nature13178 PMC4139711

[68] E. Buetas , M. Jordán‐López , A. López‐Roldán , A. Mira , and M. Carda‐Diéguez , “Impact of Periodontitis on the Leakage of Oral Bacteria to the Gut,” Journal of Dental Research 103, no. 3 (2024): 289–297. 38193290 10.1177/00220345231221709

[69] O. Koren , A. Spor , J. Felin , et al., “Human Oral, Gut, and Plaque Microbiota in Patients With Atherosclerosis,” Proceedings of the National Academy of Sciences of the United States of America 108, no. Suppl 1 (2011): 4592–4598. 20937873 10.1073/pnas.1011383107 PMC3063583

[70] E. A. Franzosa , X. C. Morgan , N. Segata , et al., “Relating the Metatranscriptome and Metagenome of the Human Gut,” Proceedings of the National Academy of Sciences of the United States of America 111, no. 22 (2014): E2329–E2338. 24843156 10.1073/pnas.1319284111 PMC4050606

[71] M. A. Jackson , J. K. Goodrich , M. E. Maxan , et al., “Proton Pump Inhibitors Alter the Composition of the Gut Microbiota,” Gut 65, no. 5 (2016): 749–756. 26719299 10.1136/gutjnl-2015-310861 PMC4853574

[72] S. Yost , A. E. Duran‐Pinedo , R. Teles , K. Krishnan , and J. Frias‐Lopez , “Functional Signatures of Oral Dysbiosis During Periodontitis Progression Revealed by Microbial Metatranscriptome Analysis,” Genome Medicine 7, no. 1 (2015): 27. 25918553 10.1186/s13073-015-0153-3 PMC4410737

[73] P. Jorth , K. H. Turner , P. Gumus , N. Nizam , N. Buduneli , and M. Whiteley , “Metatranscriptomics of the Human Oral Microbiome During Health and Disease,” MBio 5, no. 2 (2014): e01012–e01014. 24692635 10.1128/mBio.01012-14 PMC3977359

[74] S. M. Dabdoub , S. M. Ganesan , and P. S. Kumar , “Comparative Metagenomics Reveals Taxonomically Idiosyncratic Yet Functionally Congruent Communities in Periodontitis,” Scientific Reports 6, no. 1 (2016): 38993. 27991530 10.1038/srep38993 PMC5172196

[75] J. Lloyd‐Price , C. Arze , A. N. Ananthakrishnan , et al., “Multi‐Omics of the Gut Microbial Ecosystem in Inflammatory Bowel Diseases,” Nature 569, no. 7758 (2019): 655–662. 31142855 10.1038/s41586-019-1237-9 PMC6650278

[76] T. E. Van Dyke , G. Baima , and M. Romandini , “Periodontitis: Microbial Dysbiosis, Non‐Resolving Inflammation, or Both?,” Journal of Periodontal Research (2025). 10.1111/jre.13424 40657987

[77] J. Wirbel , P. T. Pyl , E. Kartal , et al., “Meta‐Analysis of Fecal Metagenomes Reveals Global Microbial Signatures That Are Specific for Colorectal Cancer,” Nature Medicine 25, no. 4 (2019): 679–689. 10.1038/s41591-019-0406-6 PMC7984229 30936547

[78] A. Barbour , O. Elebyary , N. Fine , M. Oveisi , and M. Glogauer , “Metabolites of the Oral Microbiome: Important Mediators of Multikingdom Interactions,” FEMS Microbiology Reviews 46, no. 1 (2022): fuab039. 34227664 10.1093/femsre/fuab039

[79] T. Tsuzuno , N. Takahashi , M. Yamada‐Hara , et al., “Ingestion of *Porphyromonas gingivalis* Exacerbates Colitis via Intestinal Epithelial Barrier Disruption in Mice,” Journal of Periodontal Research 56, no. 2 (2021): 275–288. 33512709 10.1111/jre.12816

[80] J. Bao , L. Li , Y. Zhang , et al., “Periodontitis May Induce Gut Microbiota Dysbiosis via Salivary Microbiota,” International Journal of Oral Science 14, no. 1 (2022): 1–11. 35732628 10.1038/s41368-022-00183-3 PMC9217941

[81] M. M. Alamri , B. Williams , A. Le Guennec , et al., “Metabolomics Analysis in Saliva From Periodontally Healthy, Gingivitis and Periodontitis Patients,” Journal of Periodontal Research 58, no. 6 (2023): 1272–1280. 37787434 10.1111/jre.13183

[82] G. Baima , G. Iaderosa , F. Citterio , et al., “Salivary Metabolomics for the Diagnosis of Periodontal Diseases: A Systematic Review With Methodological Quality Assessment,” Metabolomics 17, no. 1 (2021): 1. 33387070 10.1007/s11306-020-01754-3

[83] D. Parada Venegas , M. K. de la Fuente , G. Landskron , et al., “Short Chain Fatty Acids (SCFAs)‐Mediated Gut Epithelial and Immune Regulation and Its Relevance for Inflammatory Bowel Diseases,” Frontiers in Immunology 10 (2019): 277. 30915065 10.3389/fimmu.2019.00277 PMC6421268

[84] S. Sanna , N. R. van Zuydam , A. Mahajan , et al., “Causal Relationships Among the Gut Microbiome, Short‐Chain Fatty Acids and Metabolic Diseases,” Nature Genetics 51, no. 4 (2019): 600–605. 30778224 10.1038/s41588-019-0350-x PMC6441384

[85] A. Vrieze , E. Van Nood , F. Holleman , et al., “Transfer of Intestinal Microbiota From Lean Donors Increases Insulin Sensitivity in Individuals With Metabolic Syndrome,” Gastroenterology 143, no. 4 (2012): 913–916.e7. 22728514 10.1053/j.gastro.2012.06.031

[86] A. Agus , K. Clément , and H. Sokol , “Gut Microbiota‐Derived Metabolites as Central Regulators in Metabolic Disorders,” Gut 70, no. 6 (2021): 1174–1182. 33272977 10.1136/gutjnl-2020-323071 PMC8108286

[87] C. Pozzi , R. Levi , D. Braga , et al., “A “Multiomic” Approach of Saliva Metabolomics, Microbiota, and Serum Biomarkers to Assess the Need of Hospitalization in Coronavirus Disease 2019,” Gastro Hep Advances 1, no. 2 (2022): 194–209. 35174369 10.1016/j.gastha.2021.12.006 PMC8818445

[88] A. Hernández‐Cacho , J. F. García‐Gavilán , A. Atzeni , et al., “Multi‐Omics Approach Identifies Gut Microbiota Variations Associated With Depression,” NPJ Biofilms and Microbiomes 11, no. 1 (2025): 68. 40295565 10.1038/s41522-025-00707-9 PMC12038053

[89] C. A. Lozupone , J. I. Stombaugh , J. I. Gordon , J. K. Jansson , and R. Knight , “Diversity, Stability and Resilience of the Human Gut Microbiota,” Nature 489, no. 7415 (2012): 220–230. 22972295 10.1038/nature11550 PMC3577372

[90] M. V. Hul , P. D. Cani , C. Petitfils , W. M. D. Vos , H. Tilg , and E. M. El‐Omar , “What Defines a Healthy Gut Microbiome?,” Gut 73 (2024): 1893–1908, https://gut.bmj.com/content/73/11/1893. 39322314 10.1136/gutjnl-2024-333378 PMC11503168

[91] Y. Zhao , Y. Liu , and L. Jia , “Gut Microbial Dysbiosis and Inflammation: Impact on Periodontal Health,” Journal of Periodontal Research 60 (2025): 30–43. 38991951 10.1111/jre.13324

[92] N. Han , X. Li , J. Du , J. Xu , L. Guo , and Y. Liu , “The Impacts of Oral and Gut Microbiota on Alveolar Bone Loss in Periodontitis,” Journal of Periodontal Research 58 (2023): 1139–1147. 37712722 10.1111/jre.13168

[93] A. S. Ismail , C. L. Behrendt , and L. V. Hooper , “Reciprocal Interactions Between Commensal Bacteria and Gamma Delta Intraepithelial Lymphocytes During Mucosal Injury,” Journal of Immunology 182, no. 5 (2009): 3047–3054. 10.4049/jimmunol.0802705 PMC2763635 19234201

[94] X. Zeng , X. Wang , X. Guan , X. Feng , R. Lu , and H. Meng , “The Long‐Term Effect of Periodontitis Treatment on Changes in Blood Inflammatory Markers in Patients With Generalized Aggressive Periodontitis,” Journal of Periodontal Research 59, no. 4 (2024): 689–697. 38501229 10.1111/jre.13251

[95] Y. Wu , B. He , Q. Chen , et al., “Association Between Mediterranean Diet and Periodontitis Among US Adults: The Mediating Roles of Obesity Indicators,” Journal of Periodontal Research 59, no. 1 (2024): 32–41. 37842947 10.1111/jre.13195

[96] J. Huang , Y. Xu , and P. Huang , “Salivary miR‐150‐5p as an Indicator of Periodontitis Severity and Regulator of Human Periodontal Ligament Fibroblast Behavior by Targeting AIFM2,” Journal of Periodontal Research 59, no. 1 (2024): 187–194. 37965810 10.1111/jre.13205

