# Supplementary material for: Multi‐Omics Signatures of Periodontitis and Periodontal Therapy on the Oral and Gut Microbiome
Source: J Periodontal Res. 2025 Nov 27;60(12):1237–53. doi: 10.1111/jre.70055 (PMC12881887; doi:10.1111/jre.70055)
Supplement: Supplementary file 1 — Appendix S1: jre70055‐sup‐0001‐Appendix.docx. [file JRE-60-1237-s004.docx]

***Supplementary material***

*Supplementary Methods for Metabolomics analysis*

Frozen saliva samples were thawed at room temperature and centrifuged (18,845 RCF for 5 min at +4°C). For each saliva sample, a total of 70 μL of potassium phosphate buffer (1.5 M KH_2_PO_4_, 100% (v/v) ^2^H_2_O, 2 mM NaN_3_, 5.8 mM sodium trimethylsilyl [2,2,3,3-^2^H_4_] propionate - TMSP; pH 7.4) was added to 630 μL of sample supernatant, and the mixture was homogenized by vortexing for 30 seconds. A total of 600 μL of this mixture was transferred into a 5 mm NMR tube for analysis. For stool samples, fecal water was extracted at a ratio of 1:2.5 (g/mL, weight of feces-to buffer volume) in potassium phosphate buffer. The buffered samples were homogenized by whirl mixing for 30 s and sonicated for 15 min. Each sample was then centrifuged at 18,845 RCF for 15 min at +4°C, and 700 μL of the supernatant was stored at -80°C for NMR analysis. Frozen fecal water samples were thawed at room temperature and centrifuged (18,845 RCF for 5 min at +4°C). Subsequently, 300 μL of the supernatant was diluted with 600 μL of D_2_O, and, after 30 s of mixing, centrifuged (18,845 RCF for 10 min at +4°C). 600 μL of the supernatant was transferred into a 5 mm NMR tube for analysis.

All NMR spectra were acquired using a Bruker 600 MHz spectrometer (Bruker BioSpin) operating at 600.13 MHz proton Larmor frequency and equipped with a 5 mm PATXI ^1^H-^13^C-^15^N and ^2^H-decoupling probe including a z-axis gradient coil, an automatic tuning-matching (ATM) and an automatic refrigerated (6°C) sample changer (SampleJet, Bruker BioSpin). To ensure high spectral quality and reproducibility, the spectrometer was calibrated daily following strict standard operating procedures (including solvent suppression and temperature control). Before acquisition, samples were held in the probe for at least 5 minutes to allow them to equilibrate at the operating temperature. The Carr–Purcell–Meiboom–Gill (CPMG) one-dimensional spin–echo sequence (Bruker sequence cpmgpr1d)^34^ was applied to detect metabolite signals. For each saliva sample, the CPMG experiment was acquired at 300 K using 64 scans, 4 dummy scans, an acquisition time of 3.067 s, a relaxation delay of 4 s and a total spin-echo delay of 80 ms. For each fecal water sample, the CPMG experiment was acquired at 310 K using 128 scans, 4 dummy scans, an acquisition time of 3.067 s, a relaxation delay of 4 s and a total spin-echo delay of 80 ms. Before applying Fourier transform, free induction decays were multiplied by an exponential function equivalent to 0.3 Hz line-broadening factor. Transformed spectra were automatically corrected for phase and baseline distortions and spectra calibrated to the TMSP singlet at 0 ppm using TopSpin (version 3.6, Bruker BioSpin GmbH). Metabolites, whose peaks in the CPMG spectra were well defined and resolved, were manually assigned by using Chenomx NMR suite 12.0, freely available databases, and published literature when available. Quantification (in arbitrary units) was performed by integration using an R script in-house developed. To account for variations in water content, total spectral area normalization was applied to metabolite concentrations.
