# Supplementary figures and images for: Multi‐Omics Signatures of Periodontitis and Periodontal Therapy on the Oral and Gut Microbiome
Source: J Periodontal Res. 2025 Nov 27;60(12):1237–53. doi: 10.1111/jre.70055 (PMC12881887; doi:10.1111/jre.70055)

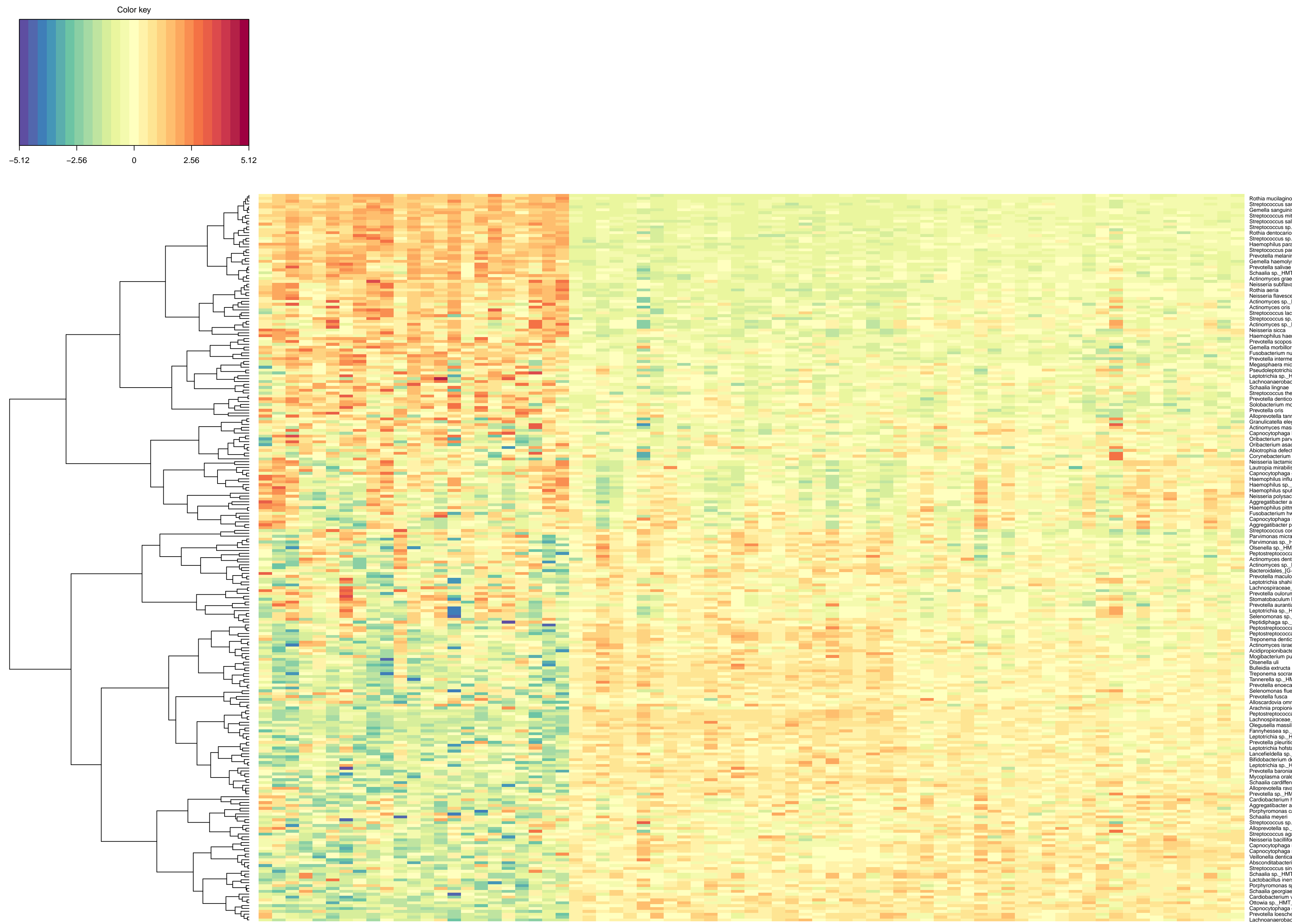

Supplement: Supplementary file 2 — Figure S1: Heatmap of species that most separate the three study groups by sparse Partial Least Squares Discriminant Analysis (sPLS‐DA) in saliva. CSaliva_T0; healthy controls, PSaliva_T0, periodontitis patients at baseline; PSaliva_T1, post‐treatment samples. [file JRE-60-1237-s005.pdf]

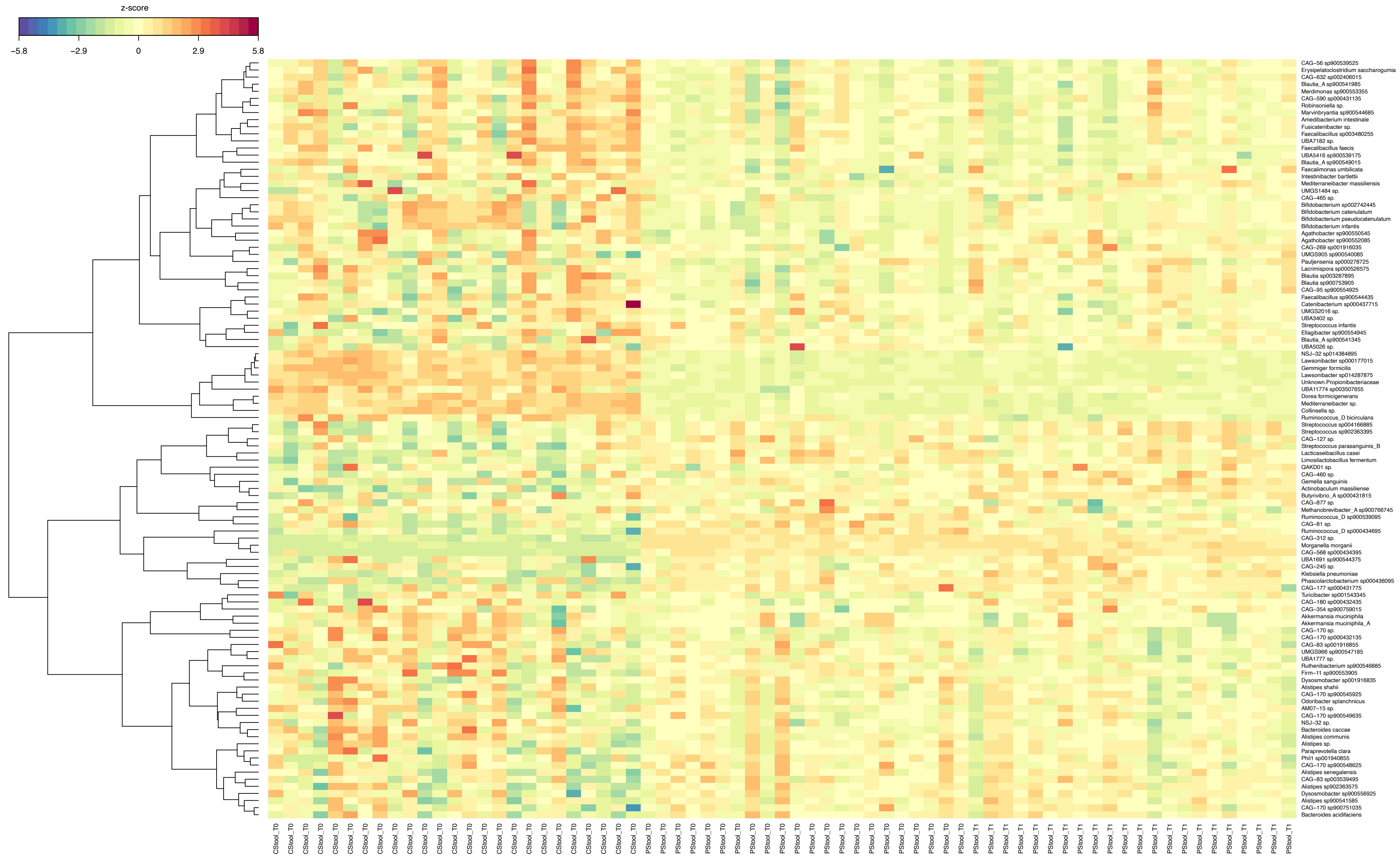

Supplement: Supplementary file 3 — Figure S2: Heatmap of species that most separate the three study groups by sparse Partial Least Squares Discriminant Analysis (sPLS‐DA) in stool. CStool_T0; healthy controls, PStool_T0, periodontitis patients at baseline; PStool_T1, post‐treatment samples. [file JRE-60-1237-s007.pdf]

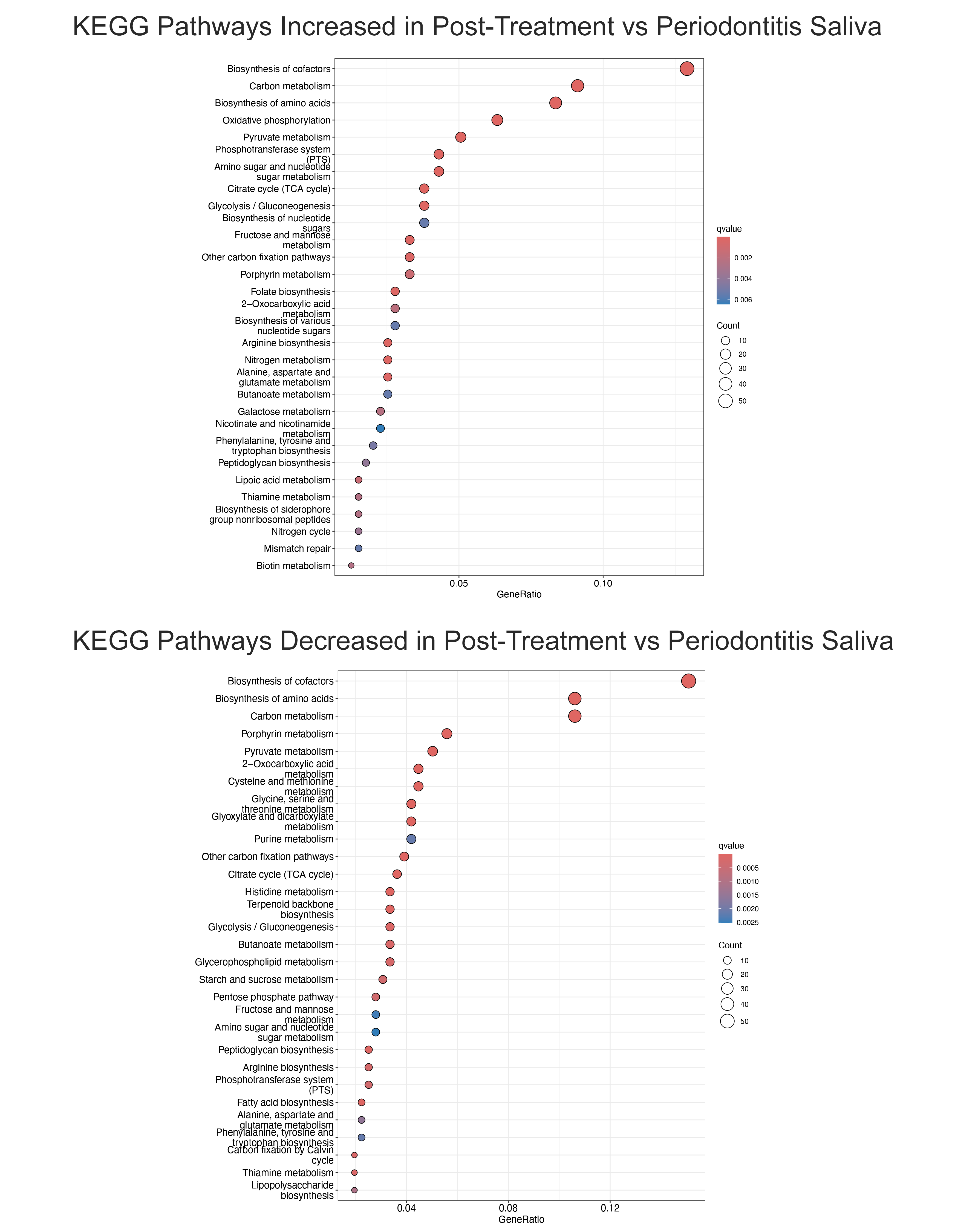

Supplement: Supplementary file 4 — Figure S3: KEGG pathway differences in salivary microbiomes between periodontitis patients pre‐ and post‐treatment. Dot plots illustrate differentially enriched microbial KEGG pathways in saliva samples from individuals with periodontitis pre‐ and post‐treatment. (Top) Pathways significantly increased in post‐treatment included central carbon metabolism, amino acid and nucleotide biosynthesis, oxidative phosphorylation, phosphotransferase systems (PTS), and various sugar and energy metabolism modules. (Bottom) Pathways reduced in post‐treatment encompassed glycan biosynthesis, lipopolysaccharide biosynthesis, butanoate metabolism, DNA repair and replication, and several cofactor and amino acid biosynthetic functions. Dot size corresponds to the number of annotated genes per pathway, and color represents adjusted q‐values. These data indicate that treatment is associated with distinct metabolic reprogramming of the salivary microbiome, reflecting increased energy generation and stress response functions alongside reductions in host‐compatible and biosynthetic pathways. [file JRE-60-1237-s003.png]

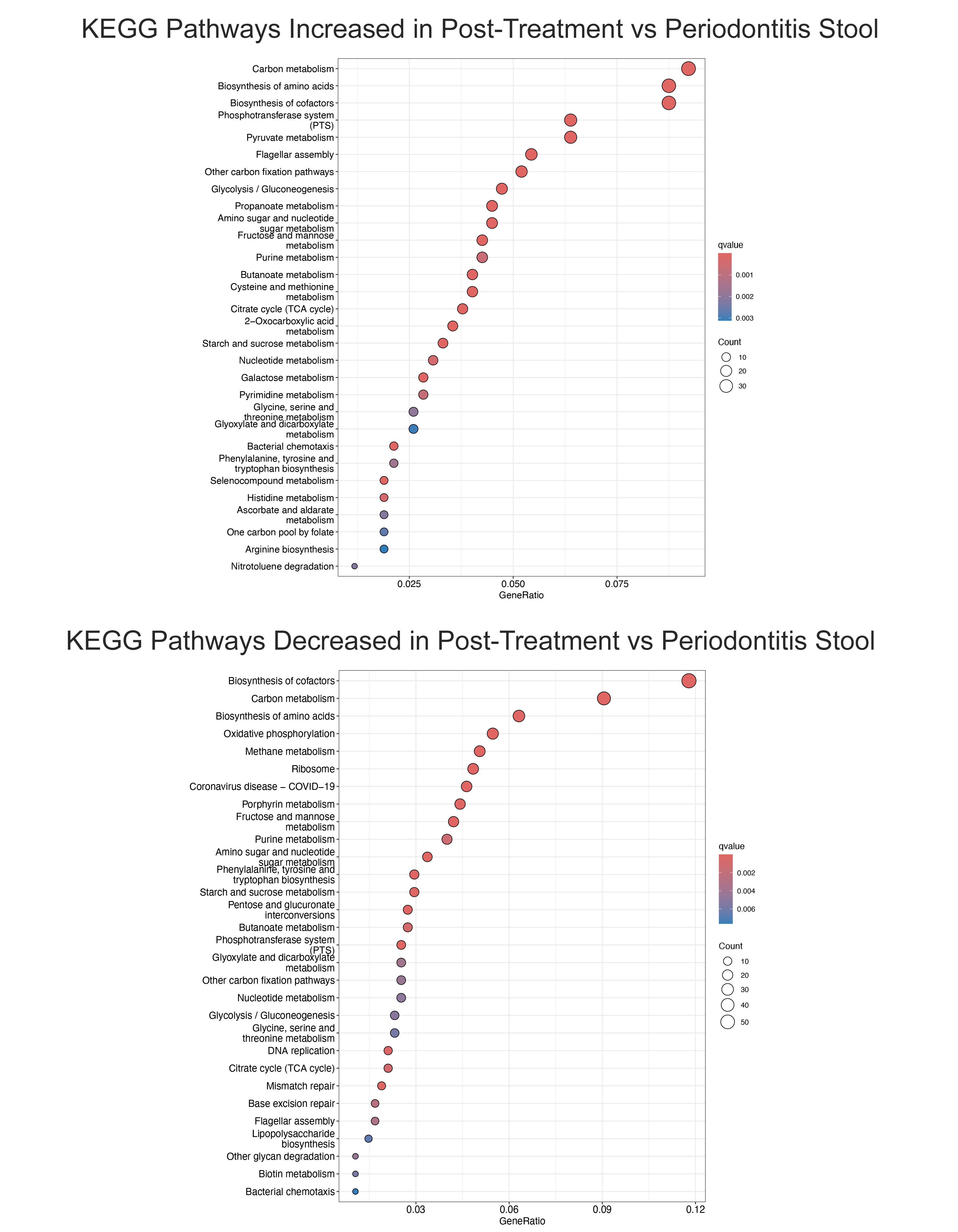

Supplement: Supplementary file 5 — Figure S4: Functional KEGG pathway differences in stool microbiomes between periodontitis patients pre‐ and post‐treatment. Dot plots display KEGG pathway enrichment analysis of microbial genes differentially abundant in stool samples from periodontitis patients pre‐ and post‐treatment. (Top) Pathways significantly enriched in post‐treatment were predominantly related to central carbon metabolism, amino acid and nucleotide metabolism, phosphotransferase systems (PTS), microbial motility (e.g., flagellar assembly, chemotaxis), and stress‐associated functions. (Bottom) Pathways decreased in post‐treatment included biosynthetic modules involved in oxidative phosphorylation, ribosome formation, biotin metabolism, glycan degradation, and multiple DNA repair and replication processes. Dot size reflects the number of genes per pathway; color denotes adjusted q‐values. These findings suggest that periodontitis treatment functionally reconfigures the gut microbiome toward energy harvesting, environmental resilience, and pro‐inflammatory potential, with depletion of host‐compatible biosynthetic and regulatory functions. [file JRE-60-1237-s008.png]
