# Supplementary material for: Multi‐Omics Signatures of Periodontitis and Periodontal Therapy on the Oral and Gut Microbiome
Source: J Periodontal Res. 2025 Nov 27;60(12):1237–53. doi: 10.1111/jre.70055 (PMC12881887; doi:10.1111/jre.70055)
Supplement: Supplementary file 6 — Table S1: jre70055‐sup‐0006‐TableS1.docx. [file JRE-60-1237-s001.docx]

**Multi-Omics Signatures of Periodontitis and Periodontal Therapy on the Oral and Gut Microbiome**

**Authors**

Giacomo Baima^1*^, Shareef Dabdoub^2,3*^, Vivek Thumbigere-Math^4^, Davide Giuseppe Ribaldone^5^, Gian Paolo Caviglia^5^, Leonardo Tenori^6^, Linda Fantato^6^, Alessia Vignoli^6^, Mario Romandini^7^, Ilario Ferrocino^8^, Mario Aimetti^1^

**Supplementary Table 1**

Dietary habits of the study population.

| **Parameters** | **Periodontitis**  **Pre-therapy** | **Periodontitis**  **Post-therapy** | **Periodontal Health** | **P-value** |
| --- | --- | --- | --- | --- |
| Carbohydrates |  |  |  | 0.173 |
| *never* | 0 (0.0%) | 1 (2.1%) | 0 (0.0%) |  |
| *sporadically* | 0 (0.0%) | 0 (0.0%) | 4 (8.5%) |  |
| *1 day/week* | 5 (10.6%) | 6 (12.8%) | 2 (4.3%) |  |
| *2-3 days/week* | 17 (36.2%) | 17 (36.2%) | 14 (29.8%) |  |
| *everyday* | 25 (53.2%) | 23 (48.9%) | 27 (57.4%) |  |
| Legumes |  |  |  | 0.300 |
| *never* | 6 (12.8%) | 4 (8.5%) | 4 (8.5%) |  |
| *sporadically* | 6 (12.8%) | 7 (14.9%) | 13 (27.7%) |  |
| *1 day/week* | 25 (53.2%) | 21(44.7%) | 22 (46.8%) |  |
| *2-3 days/week* | 8 (17.0%) | 15 (31.9%) | 7 (14.9%) |  |
| *everyday* | 2 (4.3%) | 0 (0.0%) | 1 (2.1%) |  |
|  |  |  |  |  |
| Animal proteins |  |  |  | 0.775 |
| *never* | 3 (6.4%) | 3 (6.4%) | 0 (0.0%) |  |
| *sporadically* | 4 (8.5%) | 3 (6.4%) | 4 (8.5%) |  |
| *1 day/week* | 9 (19.1%) | 11 (23.4%) | 13 (27.7%) |  |
| *2-3 days/week* | 22 (46.8%) | 24 (51.1%) | 22 (46.8%) |  |
| *everyday* | 9 (19.1%) | 6 (12.8%) | 8 (17.0%) |  |
| Fruit |  |  |  |  |
| *never* | 1 (2.1%) | 1 (2.1%) | 1 (2.1%) | 0.972 |
| *sporadically* | 1 (2.1%) | 1 (2.1%) | 3 (6.4%) |  |
| *1 day/week* | 5 (10.6%) | 8 (17.0%) | 6 (12.8%) |  |
| *2-3 days/week* | 8 (17.0%) | 6 (12.8%) | 7 (14.9%) |  |
| *everyday* | 32 (68.1%) | 31 (66.0%) | 30 (63.8%) |  |
|  |  |  |  |  |
| Vegetables |  |  |  | 0.152 |
| *never* | 0 (0.0%) | 0 (0.0%) | 0 (0.0%) |  |
| *sporadically* | 1 (2.1%) | 1 (2.1%) | 0 (0.0%) |  |
| *1 day/week* | 3 (6.4%) | 5 (10.6%) | 0 (0.0%) |  |
| *2-3 days/week* | 15 (31.9%) | 9 (19.1%) | 14 (29.8%) |  |
| *everyday* | 28 (59.6%) | 32 (68.1%) | 33 (70.2%) |  |
|  |  |  |  |  |
| Dairy products |  |  |  | 0.329 |
| *never* | 0 (0.0%) | 3 (6.4%) | 1 (2.1%) |  |
| *sporadically* | 3 (6.4%) | 5 (10.6%) | 5 (10.6%) |  |
| *1 day/week* | 16 (34.0%) | 14 (29.8%) | 8 (17.0%) |  |
| *2-3 days/week* | 12 (25.5%) | 9 (19.1%) | 17 (36.2%) |  |
| *everyday* | 16 (34.0%) | 16 (34.0%) | 16 (34.0%) |  |
|  |  |  |  |  |
| Eggs |  |  |  | 0.986 |
| *never* | 2 (4.3%) | 2 (4.3%) | 2 (4.3%) |  |
| *sporadically* | 13 (27.7%) | 16 (34.0%) | 12 (25.5%) |  |
| *1 day/week* | 25 (53.2%) | 22 (46.8%) | 26 (55.3%) |  |
| *2-3 days/week* | 7 (14.9%) | 6 (12.8%) | 7 (14.9%) |  |
| *everyday* | 0 (0.0%) | 1 (2.1%) | 0 (0.0%) |  |
| Alcohol |  |  |  | 0.174 |
| *never* | 25 (53.2%) | 25 (53.2%) | 15 (31.9%) |  |
| *sporadically* | 9 (19.1%) | 12 (25.5%) | 19 (40.4%) |  |
| *1 day/week* | 6 (12.8%) | 2 (4.3%) | 5 (10.6%) |  |
| *2-3 days/week* | 3 (6.4%) | 6 (12.8%) | 6 (12.8%) |  |
| *everyday* | 4 (8.5%) | 2 (4.3%) | 2 (4.3%) |  |
